# Using a novel source-localized phase regressor technique for evaluation of the vascular contribution to semantic category area localization in BOLD fMRI

**DOI:** 10.3389/fnins.2015.00411

**Published:** 2015-11-03

**Authors:** An T. Vu, Jack L. Gallant

**Affiliations:** ^1^Program in Bioengineering, University of California, BerkeleyBerkeley, CA, USA; ^2^Helen Wills Neuroscience Institute, University of California, BerkeleyBerkeley, CA, USA; ^3^Department of Psychology, University of California, BerkeleyBerkeley, CA, USA

**Keywords:** vein suppression, Fusiform Face Area (FFA), Parahippocampal Place Area (PPA), phase, complex valued, BOLD fMRI

## Abstract

Numerous studies have shown that gradient-echo blood oxygen level dependent (BOLD) fMRI is biased toward large draining veins. However, the impact of this large vein bias on the localization and characterization of semantic category areas has not been examined. Here we address this issue by comparing standard magnitude measures of BOLD activity in the Fusiform Face Area (FFA) and Parahippocampal Place Area (PPA) to those obtained using a novel method that suppresses the contribution of large draining veins: source-localized phase regressor (sPR). Unlike previous suppression methods that utilize the phase component of the BOLD signal, sPR yields robust and unbiased suppression of large draining veins even in voxels with no task-related phase changes. This is confirmed in ideal simulated data as well as in FFA/PPA localization data from four subjects. It was found that approximately 38% of right PPA, 14% of left PPA, 16% of right FFA, and 6% of left FFA voxels predominantly reflect signal from large draining veins. Surprisingly, with the contributions from large veins suppressed, semantic category representation in PPA actually tends to be lateralized to the left rather than the right hemisphere. Furthermore, semantic category areas larger in volume and higher in fSNR were found to have more contributions from large veins. These results suggest that previous studies using gradient-echo BOLD fMRI were biased toward semantic category areas that receive relatively greater contributions from large veins.

## Introduction

Studies using BOLD fMRI rely on the assumption that differences in strength of task-related BOLD activity reflect differences in strength of underlying neural activity. Comparisons of BOLD activity to neural activity in individual regions of cortex have warranted this assumption (for review see Logothetis and Wandell, 2004). However, studies on the representation of semantic categories often assume that BOLD fMRI can be used to infer neural differences across cortical regions and across subjects. However, given the bias of BOLD fMRI toward large veins, BOLD activity differences across cortical regions and across subjects may simply reflect differences in vein size (Lai et al., 1993; Haacke et al., 1994; Kim et al., 1996; Jochimsen et al., 2004).

In spite of the bias toward large veins, fMRI studies comparing BOLD activity across cortical regions have strongly shaped our understanding of semantic representation in the human brain. Early studies suggested that distinct areas of cortex are responsible for the representation of specific semantic categories: faces (Kanwisher et al., 1997), places (Epstein and Kanwisher, 1998), and body parts (Downing et al., 2001). However, areas selective for other semantic categories have yet to be discovered and much of visual cortex remains to be functionally characterized (Downing et al., 2006; Reddy and Kanwisher, 2006; Spiridon et al., 2006; however see Huth et al., 2012). These results led to the hypothesis that representation of other semantic categories are not represented in cortical areas but by the fine scaled pattern of activity across cortex (Haxby et al., 2001; Ewbank et al., 2005). However, because gradient-echo BOLD fMRI is biased toward large veins, the rarity of semantic category areas could simply reflect a bias in the distribution of vein size toward specific regions of interest (ROIs).

Studies using gradient-echo BOLD fMRI have also suggested that semantic category representation is right hemisphere lateralized (Kanwisher et al., 1997; Epstein and Kanwisher, 1998; Downing et al., 2006; Spiridon et al., 2006). In these studies, the right Parahippocampal Place Area (PPA) tends to be larger in size and higher in functional SNR (fSNR, measured using appropriate *t*-value contrasts) than left PPA. The Fusiform Face Area (FFA) tends to be even more right hemisphere lateralized than PPA. In addition to finding right FFA larger in size and higher in fSNR than left FFA, for some subjects the left FFA is not found at all (Kanwisher et al., 1997; Yovel and Kanwisher, 2004; Spiridon et al., 2006). These results suggest that semantic representation in the human brain is heavily right hemisphere lateralized. However, because BOLD fMRI is biased toward large veins, the laterality of semantic representation may also reflect the general trend that veins are larger in the right hemisphere (Di Chiro, 1972; Durgun et al., 1993; Ayanzen et al., 2000; Stoquart-ElSankari et al., 2009). If true, ROIs larger in size and higher in fSNR will have larger contributions from large veins.

To evaluate this hypothesis, we first develop a novel method for suppressing the large vein contribution to BOLD activity: source-localized phase regressor (sPR). We then measure ROI size and fSNR with and without large vein suppression to quantify the amount of large vein contribution to individual ROIs. sPR builds upon the phase regressor (PR) large vein suppression method developed by Menon (2002) with two key improvements (see Large vein suppression methods for details). First, sPR utilizes an unbiased least squares loss function to avoid the overcorrection of large vein contributions of PR as reported by Nencka and Rowe (2007). Second, sPR utilizes task-related phase changes in neighboring voxels to suppress large vein contributions even in voxels with poor phase fSNR. Thus, sPR is able to suppress large veins roughly the size of a voxel, oriented at any arbitrary angle including those near the magic angle (i.e., the angle, ~54°, between a vein and B_0_ which results in zero intravascular phase accrual; Reichenbach et al., 2000; Menon, 2002). Together, these improvements allow sPR to yield robust, unbiased suppression of large vein contributions even in voxels with little or no task-related phase changes.

The first portion of this study validates the sPR method by comparing it to the original PR method under various simulated magnitude and phase fSNR conditions. Through this simulation it is demonstrated that sPR yields robust and unbiased suppression of large vein contributions. The second portion of this study compares standard magnitude measures of BOLD activity in FFA and PPA to those obtained using sPR large vein suppression. Large vein contributions are then quantified in individual ROIs to reveal the relationship between ROI size, fSNR, and large vein contributions. How these results affect interpretation of BOLD activity measured using gradient-echo BOLD fMRI is also discussed.

## Large vein suppression methods

**Figure 1:**
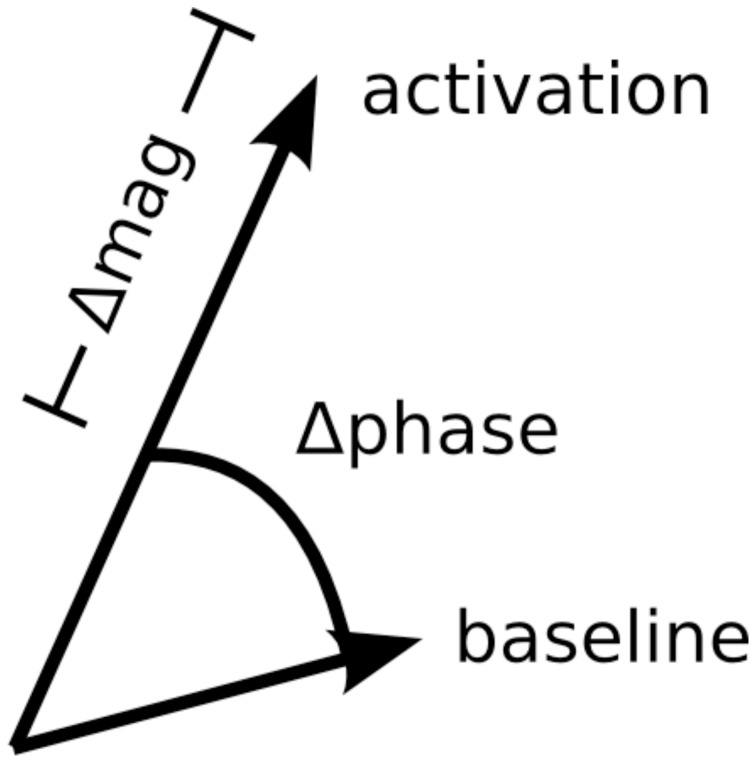
**Diagram of task-related phase and magnitude changes in the complex plane**.

Previous studies have developed methods for suppressing large vein contributions to BOLD activity using standard gradient-echo pulse sequences (Menon, 2002; Nencka and Rowe, 2007). In contrast to most BOLD fMRI studies that only use the magnitude component of the BOLD signal, these large vein suppression methods utilize the entire complex-valued BOLD signal: both magnitude and phase components (Figure 1). Taking note that task-related phase changes come exclusively from large veins, these large vein suppression techniques penalize task-related magnitude changes based on the amount of task-related phase changes in a given voxel. These techniques are attractive because they do not require pulse sequence modification or reductions in spatial/temporal resolution. However, Nencka and Rowe (2007) cautioned against the use of these large vein suppression techniques given their tendency to over- or under-correct for large vein contributions. Furthermore, these large vein suppression techniques assume that voxels containing large veins will have high phase fSNR. This assumption begins to breakdown for large veins roughly the size of a voxel as well as for veins near the magic angle (Reichenbach et al., 2000; Menon, 2002). Because these large vein suppression methods depend on task-related phase changes, voxels with low phase fSNR will be poorly suppressed even if they contain strong magnitude fSNR from large veins.

To address these limitations, we developed an unbiased and robust method for large vein suppression based on the phase regressor (PR) method of Menon (2002). As in Menon (2002), the large vein suppressed microvascular BOLD signal, *S*^*mirco*^, is defined as the magnitude component of the BOLD signal, *S*^*m*^, minus the large vein macrovascular BOLD signal, *S*^*macro*^. Thus, *S*^*macro*^can be mathematically expressed as:

(1) $$\begin{array}{l} S^{micro}=S^{m}-S^{macro}. \end{array}$$

*S*^*macro*^ is defined as the linear least-squares fit of the phase component of the BOLD signal, *S*^*p*^, to the magnitude component, *S*^*m*^. Mathematically, *S*^*macro*^can be expressed as:

(2) $$\begin{array}{l} S^{\text{m}acro}=b_{0}^{*}+b_{1}^{*}S^{p} \end{array}$$

where

(3) $$\begin{array}{l} b_{0}^{*},b_{1}^{*}=\text{arg }\underset{b_{0},b_{1}}{\min}L\;\left(b_{0},b_{1}\right). \end{array}$$

In Menon (2002) the chi squared loss function was used:

(4) $$\begin{array}{l} L_{ChiSq}\left(b_{0,}b_{1}\right)\;=\;\overset{N}{\underset{t=1}{\sum }}{\left(S^{m}\left(t\right)-b_{0}-b_{1}S^{p}\left(t\right)\right)}^{2}∕\left(\sigma_{m}^{2}+b_{1}\sigma_{p}^{2}\right) \end{array}$$

Minimizing *L*_*ChiSq*_ with respect to *b*_0_ and *b*_1_ yields the PR measure of BOLD activity for the *i*th voxel at time t given by:

(5) $$\begin{array}{l} S_{i}^{PR}\left(t\right)=S_{i}^{m}\left(t\right)-b_{1}^{*}S_{i}^{p}\left(t\right) \end{array}$$

where

(6) $$\begin{array}{l} b_{1}^{*}=-B+\sqrt{\left(B^{2}-4\text{AC}\right)}∕\left(2\text{A}\right), \end{array}$$

(7) $$\begin{array}{l} A=r\sum {\left(S^{m}\right)}^{2}∕2, \end{array}$$

(8) $$\begin{array}{l} B=\sum {\left(S^{p}\right)}^{2}-r\sum S^{m}, \end{array}$$

(9) $$\begin{array}{l} C=-\left(\sum \left(S^{p}S^{m}\right)+r\sum {\left(S^{m}\right)}^{2}\right)∕2, \end{array}$$

and

(10) $$\begin{array}{l} r=\text{sign}\left(\text{corr}\left(S^{m},S^{p}\right)\right). \end{array}$$

*S*^*m*^ and *S*^*p*^ are the z-scored magnitude and phase components of the complex-valued BOLD signal, respectively. *corr*(*S*^*m*^, *S*^*p*^) is the temporal correlation between *S*^*m*^ and*S*^*p*^ and is calculated from a separate run in the FFA/PPA localization experiment. The PR method derived here is identical to the method used in Menon (2002) except that a linear (as opposed to quartic) *b*_1_ term is used in the denominator of the loss function. This allows us to express $b_{1}^{*}$ in closed form. However, because *L*_*ChiSq*_ includes *b*_1_ in the denominator, $b_{1}^{*}$ and therefore *S*^*macro*^ tends to be overestimated (which results in over-suppression of large veins and suppression even in cases where no large veins exist). To address this, instead of *L*_*ChiSq*_, the proposed method, sPR, uses the unbiased ordinary least squares loss function:

(11) $$\begin{array}{l} L_{OLS}\left(b_{0,}b_{1}\right)=\overset{N}{\underset{t=1}{\sum }}{\left(S^{m}\left(t\right)-b_{0}-b_{1}S^{p}\left(t\right)\right)}^{2}. \end{array}$$

Because *L*_OLS_ does not include *b*_1_ in the denominator, it does not overestimate *S*^*macro*^.

**Figure 2:**
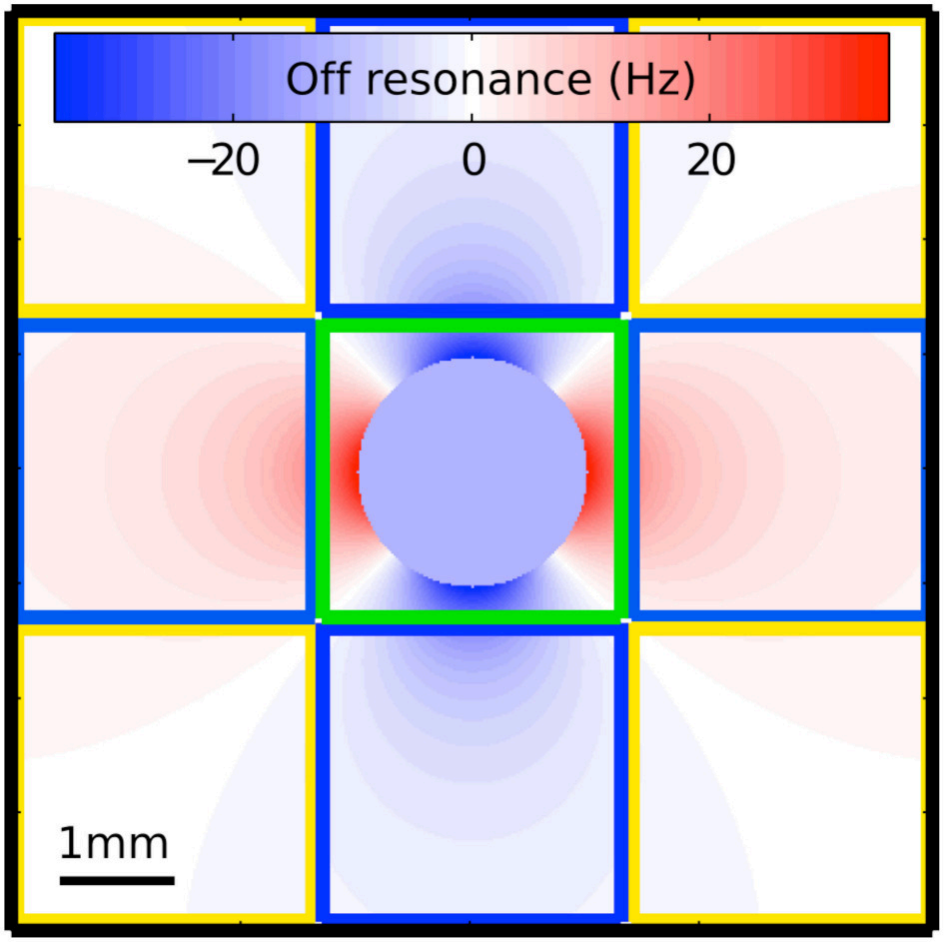
**Magnetic field off resonance distribution around the cross section of a large vein perpendicular to the main magnetic field**. This simulation uses the venogram scan parameters in our study and assumes a typical blood oxygenation fraction of 0.54 (Haacke et al., 1995). The voxel outlined in green covers the entire cross section of the vein but samples only a limited portion of the off resonance distribution. This voxel is expected to have high magnitude fSNR but low phase fSNR because the off resonance field distortions sampled by it vary symmetrically around 0 Hz and the T2^*^ of venous blood is short relative to that of the parenchyma. The voxels outlined in blue sample from the field distribution adjacent to the vein. These voxels have low magnitude fSNR but high phase fSNR because the field distortions sampled by them vary weakly and tend to be of a single polarity. The voxels outlined in yellow sample portions of the field distribution that are mostly on resonance. These voxels have very little magnitude and phase fSNR.

To address the poor suppression of large veins roughly the size of a voxel and those near the magic angle, sPR takes advantage of the magnitude and phase fSNR distribution around such veins (Figure 2). Voxels that contain a large vein roughly the size of a voxel (as the one outlined in green) have high magnitude but low phase fSNR. This is because the off resonance field distortions sampled by these voxels vary symmetrically around 0 Hz. Furthermore, intravascular task-related phase changes in these voxels, especially at higher field strengths (Yacoub et al., 2001; Duong et al., 2003), will contribute minimally due to the short T2^*^ of venous blood relative to that of the parenchyma (Reichenbach et al., 1998). Thus, using the weak task-related phase changes in these voxels often yields poor suppression. However, voxels adjacent to veins (as the ones outlined in blue in Figure 2) have low magnitude but high phase fSNR. This is because the field distortions sampled by these voxels vary weakly and tend to be of a single polarity. By using the high phase fSNR from these vein-adjacent voxels, sPR can correctly suppress BOLD activity from large vein containing voxels with poor phase fSNR. It is because veins are the source of the high phase fSNR in the vein-adjacent voxels that we call this novel technique: source-localized phase regressor (sPR). Note that while Figure 2 was generated for the case of a vein perpendicular to B_0_, the shape of the magnetic field off resonance generalizes to veins of all orientations—except for those in parallel with B_0_, in which case the extravascular frequency shifts are zero (Ogawa et al., 1993a,b). For veins more in parallel with B_0_, the sPR method will perform the same as the PR method, given the same loss function.

Minimizing *L*_*OLS*_ with respect to *b*_0_ and *b*_1_ yields the sPR measure of BOLD activity for the *i*th voxel at time *t* given by:

(12) $$\begin{array}{l} S_{i}^{sPR}\left(t\right)=S_{i}^{m}\left(t\right)-\text{corr}\left(S_{i}^{m},S_{k^{*}}^{p}\right)S_{k^{*}}^{p}\left(t\right) \end{array}$$

where k^*^ is the index of the voxel whose phase component is most correlated with the magnitude component of voxel *i*. In the simulation study, k^*^ is set to *i* (a one-voxel neighborhood) so that the difference in loss function between PR and sPR is directly compared. However, in the FFA/PPA localization study, k^*^ is allowed to be within the seven-voxel neighborhood of voxels including the six face-adjacent voxels (in 3D space) and voxel *i*. For proof of concept, this seven voxel neighborhood was chosen as the smallest, most logical increment above a one voxel neighborhood of the PR method. Future studies are necessary to determine the optimality of choosing a single most corelated voxel from larger neighborhoods vs. kernel weighted neighborhoods.

## Computer simulation study

To verify that sPR suppresses BOLD activity from large veins without overcorrection, here we compare PR and sPR suppression of simulated complex-valued BOLD activity. The Matlab code used to perform the simulations can be found in the Supplementary Materials as well as https://github.com/gallantlab/sPR_simulation_code.

### Methods

Simulated BOLD activity was generated with 14 alternating 16 s stimulus-off and stimulus-on blocks using Matlab (The Mathworks, Natick, MA). For simplicity, the hemodynamic response function was modeled as a unit impulse function with zero delay. The expected fSNR of both magnitude and phase timecourses was varied from 0 to 10 in steps of 0.1. Expected fSNR is defined as: (μ_*on*_ − μ_*off*_) ∕ σ_*noise*_, where μ_*on*_ and μ_*off*_ are the expected responses to stimulus-on and stimulus-off conditions, respectively and σ_*noise*_ is the expected standard deviation of the normally distributed temporal noise. For each method of suppression, one simulation was done for each pair of magnitude and phase fSNR values yielding a total of 2 × 101 × 101 = 20,402 simulations. fSNR of the simulated large vein suppressed BOLD activity was calculated as $\left({\bar{S}}_{on}-{\bar{S}}_{off}\right)∕\left(\text{std}\left(S_{on}\right)∕2+\text{std}\left(S_{off}\right)∕2\right)$, where ${\bar{S}}_{on}$ and ${\bar{S}}_{off}$ are the mean responses to stimulus-on and stimulus-off conditions, respectively. Averaging across repeated simulations was not done to preserve the random effect of noise under low fSNR conditions.

### Results

**Figure 3:**
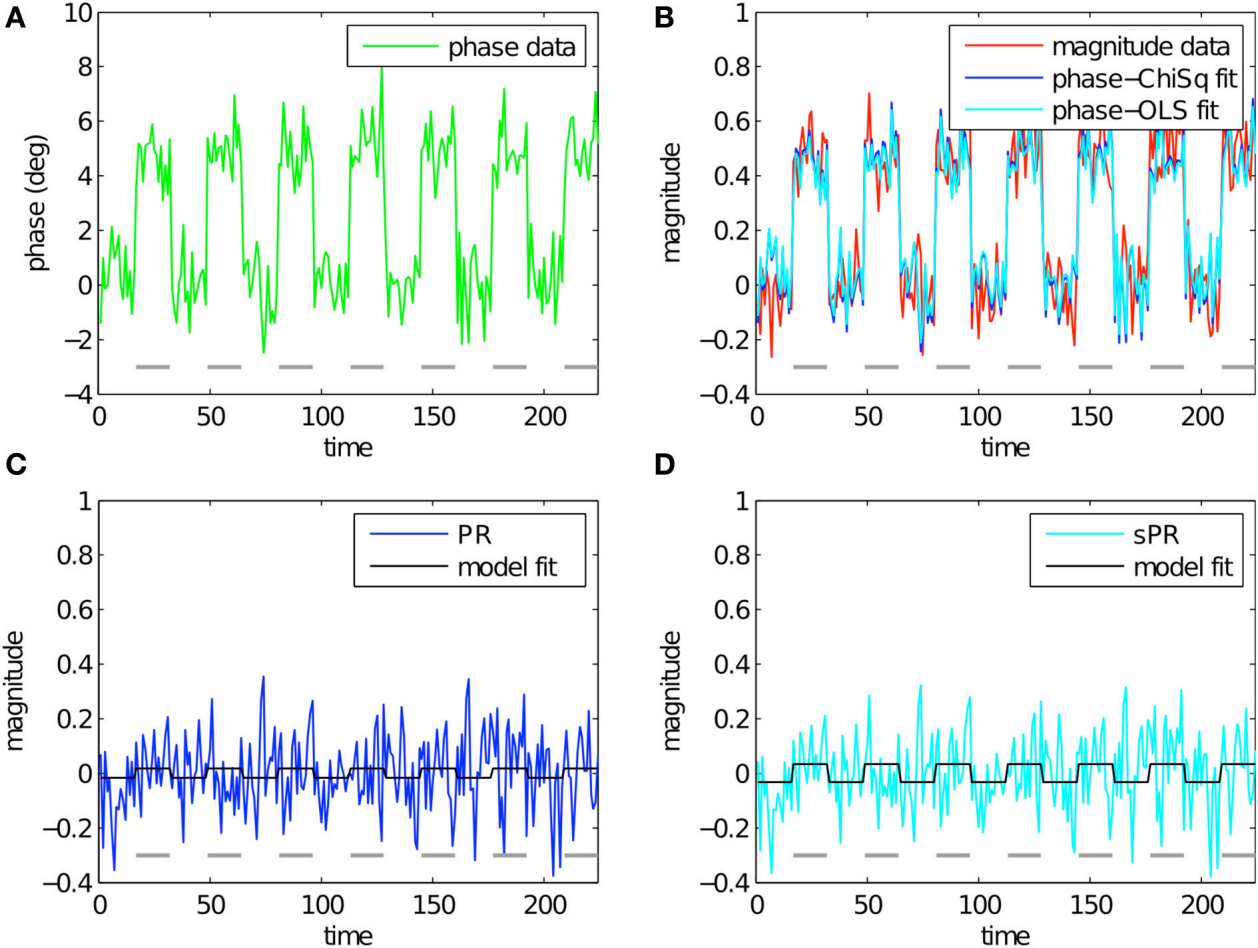
**Simulated large vein suppression in a voxel containing a large vein**. In this regime, both phase, and magnitude fSNR are high. **(A)** Phase timecourse is plotted in green (*t* = 5.16). Gray dashes indicate stimulus-on periods. **(B)** Magnitude timecourse is plotted in red (*t* = 5.08). Linear fits of phase to magnitude using *L*_*ChiSq*_ and *L*_*OLS*_ are plotted in blue and cyan, respectively. Using *L*_*ChiSq*_ yields larger absolute fits (e.g., larger $b_{1}^{*}$ values). **(C)** PR large vein suppressed timecourse is plotted in blue (*t* = 0.25). PR model fit timecourse is plotted in black. **(D)** sPR large vein suppressed timecourse is plotted in cyan (*t* = 0.50). sPR model fit timecourse is plotted in black. In this regime, both PR and sPR show good suppression of BOLD activity. PR exhibits slightly stronger suppression than sPR.

To study the effect of loss function on large vein suppression, we compare PR and sPR suppression of simulated complex-valued BOLD activity. This was done for various magnitude and phase fSNR conditions. Figure 3 shows the simulation of large vein suppression in a voxel containing a large vein. In this regime, both the phase and magnitude fSNR are high (*t* = 5.16, *t* = 5.08, respectively; Figures 3A,B). The linear fits of phase to magnitude using *L*_*ChiSq*_ (blue) and *L*_*OLS*_ (cyan) loss functions are plotted in Figure 3B. Using *L*_*ChiSq*_ yields larger absolute fits (e.g., larger $b_{1}^{*}$ values). In this regime, both PR (blue, Figure 3C), and sPR (cyan, Figure 3D) yield good suppression of BOLD activity. However, PR exhibits slightly stronger suppression than sPR as expected given its tendency of *L*_*ChiSq*_ to overestimate $b_{1}^{*}$ (*t* = 0.25 vs. *t* = 0.50, respectively).

**Figure 4:**
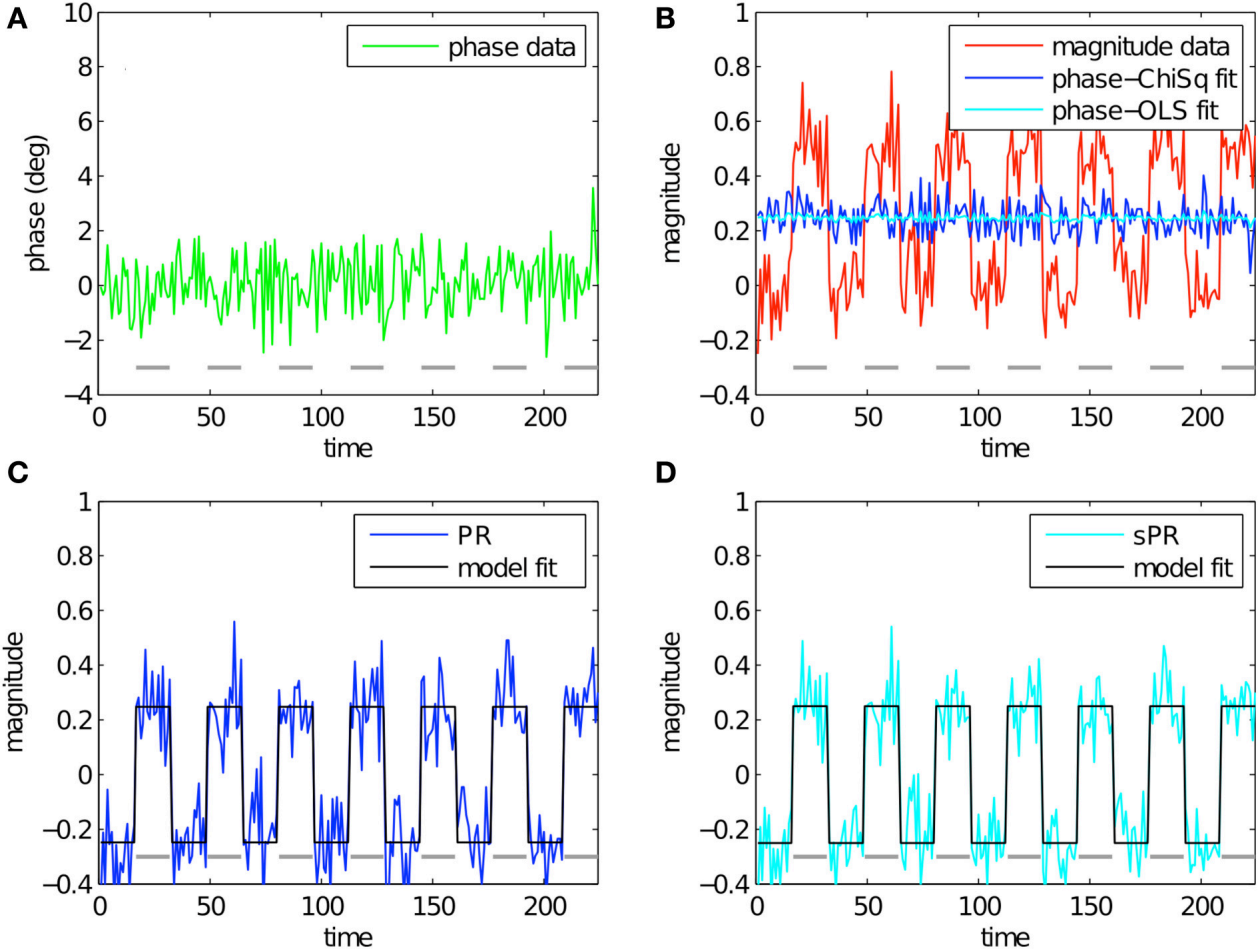
**Simulated large vein suppression in a voxel with no large vein contribution**. In this regime, phase fSNR is low but magnitude fSNR is high. **(A)** Phase timecourse is plotted in green (*t* = −0.10). Gray dashes indicate stimulus-on periods. **(B)** Magnitude timecourse is plotted in red (*t* = 5.21). Linear fits of phase to magnitude using *L*_*ChiSq*_ and *L*_*OLS*_ are plotted in blue and cyan, respectively. Using *L*_*ChiSq*_ yields larger absolute fits (e.g., larger $b_{1}^{*}$ values). **(C)** PR large vein suppressed timecourse is plotted in blue (*t* = 4.38). PR model fit timecourse is plotted in black. **(D)** sPR large vein suppressed timecourse is plotted in cyan (*t* = 5.16). sPR model fit timecourse is plotted in black. In this regime, both PR and sPR show good preservation of magnitude BOLD activity. sPR exhibits slightly better preservation than PR.

Figure 4 shows the simulation of large vein suppression in a voxel with no large vein contribution. In this regime, phase fSNR is low while magnitude fSNR is high (*t* = −0.10, *t* = 5.21, respectively; Figures 4A,B). The linear fits of phase to magnitude using *L*_*ChiSq*_ (blue) and *L*_*OLS*_ (cyan) loss functions are plotted in Figure 4B. Using *L*_*ChiSq*_ in this regime also yields larger absolute fits (e.g., larger $b_{1}^{*}$ values). Both PR (blue, Figure 4C) and sPR (cyan, Figure 4D) yield good preservation of the magnitude BOLD activity. This is the desired effect in this regime because there is no large vein contribution. However, as expected given the tendency of *L*_*ChiSq*_ to overestimate $b_{1}^{*}$, PR does exhibit some unwanted suppression in the form of enhanced noise and lower *t*-values while sPR does not (*t* = 4.38 vs. *t* = 5.16, respectively).

**Figure 5:**
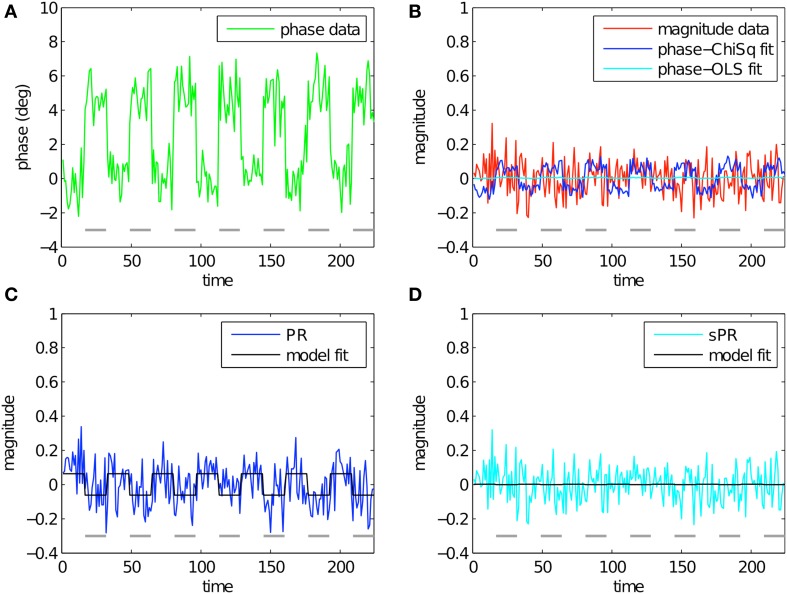
**Simulated large vein suppression in a voxel adjacent to a large vein**. In this regime, phase fSNR is high but magnitude fSNR is low. **(A)** Phase timecourse is plotted in green (*t* = 4.55). Gray dashes indicate stimulus-on periods. **(B)** Magnitude timecourse is plotted in red (*t* = 0.02). Linear fits of phase to magnitude using *L*_*ChiSq*_ and *L*_*OLS*_ are plotted in blue and cyan, respectively. Using *L*_*ChiSq*_ yields larger absolute fits (e.g., larger $b_{1}^{*}$ values). **(C)** PR large vein suppressed timecourse is plotted in blue (*t* = −1.22). PR model fit timecourse is plotted in black. **(D)** sPR large vein suppressed timecourse is plotted in cyan (*t* = −0.02). sPR model fit timecourse is plotted in black. In this regime, PR overestimates $b_{1}^{*}$ resulting in artifactual BOLD activity. sPR yields unbiased estimates of $b_{1}^{*}$ and correctly estimates the large vein suppressed BOLD timecourse as the magnitude timecourse.

Figure 5 shows the simulation of large vein suppression in a voxel adjacent to a large vein. In this regime, phase fSNR is high while magnitude fSNR is low (*t* = 4.55, *t* = 0.02, respectively; Figures 5A,B). Using *L*_*ChiSq*_ in this regime clearly results in overestimation of $b_{1}^{*}$ (blue, Figure 5B). Because in this simulation both magnitude and phase *t*-values are positive, the overestimation of $b_{1}^{*}$ generates artifactual negative BOLD activity (blue, *t* = −1.22, Figure 5C). Using *L*_*OLS*_ does not result in overestimation of $b_{1}^{*}$ (cyan, Figure 5B). Instead, sPR yields a large vein suppresed timecourse almost identical to the magnitude timecourse (cyan, *t* = −0.02, Figure 5D). In this regime, this is the desired effect because there is little task-related magnitude BOLD activity to suppress.

**Figure 6:**
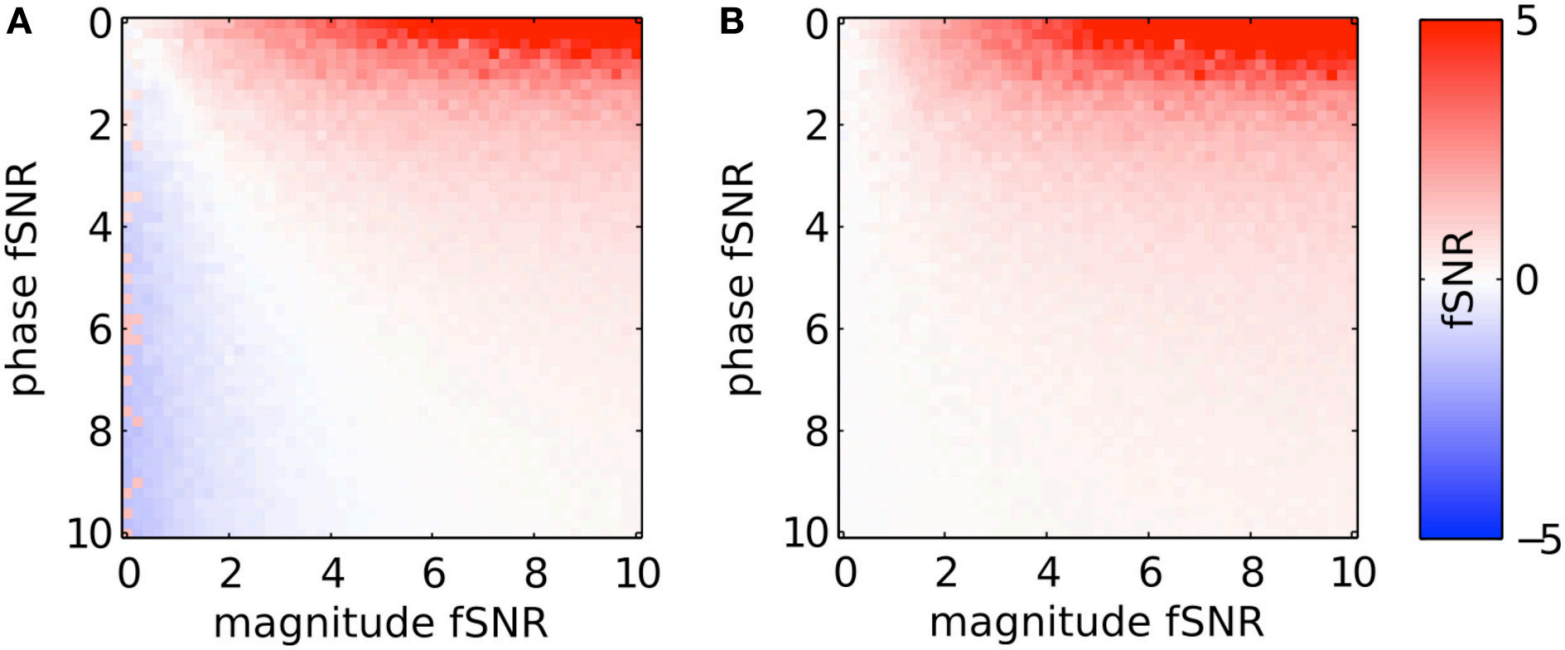
**Simulated large vein suppression as a function of phase and magnitude fSNR plotted for (A) PR and (B) sPR**. PR overcorrects for large vein contributions. This results in artifactual non-zero *t*-values at low magnitude fSNRs. sPR does not overcorrect for large vein contributions.

To generalize the above results, Figure 6 shows the resulting fSNR (as defined at the beginning of the Methods Section) after applying PR and sPR on simulated complex-valued BOLD activity under the full range of magnitude and phase fSNR conditions. Under the conditions when large veins contribute to BOLD activity (high phase, high magnitude fSNR conditions) both PR and sPR yield robust suppression of fSNR (Figures 6A,B). Under the conditions where large veins may not be contributing to BOLD activity (low phase fSNR conditions) both sPR and, to a lesser degree, PR preserve magnitude fSNR. However, under the conditions when a voxel is adjacent to a large vein (high phase, low magnitude fSNR conditions) PR overcorrects yielding artifactual positive or negative fSNR (Figure 6A). sPR does not exhibit this overcorrection (Figure 6B). These results demonstrate that sPR provides robust and unbiased suppression without introducing artifactual BOLD activity. Given these results, we use sPR to suppress large vein contributions to BOLD activity in the subsequent FFA/PPA localization experiment.

## FFA/PPA localization study

Here we invesigate the impact of large vein bias on the localization and characterization of semantic category areas. Standard magnitude measures of BOLD activity used in localizing FFA and PPA are compared to those obtained using sPR large vein suppression. Through this study, the large vein contributions to individual ROIs are quantified and the relationship between ROI size, fSNR and large vein contributions are evaluated.

### Methods

All MR data were collected with a whole-head volume radio frequency coil on the 4 Tesla Varian (Palo Alto, CA) INOVA scanner at UC Berkeley and reconstructed using Recon Tools written by the Berkeley Imaging Center. Procedures were approved by the UC Berkeley Committee for the Protection for Human Subjects and performed on four subjects (all male, mean age 28) who gave written informed consent.

#### FFA/PPA localization

FFA and PPA were localized using standard methods (Spiridon et al., 2006). The stimuli consisted of images from three categories: inanimate objects, human faces, and places. Each category contained 20 colored exemplars downloaded from the public Caltech 256 database (http://www.vision.caltech.edu/Image_Datasets/Caltech256/). Stimuli were presented at 500 × 500 pixels, extending across 20° of the visual field. A white fixation point was visible at all times.

All subjects were scanned for two 384 s runs. The first run was set aside and used only to estimate the temporal correlation between magnitude and phase components. These temporal correlations were then applied to the responses of the second run for calculation of the sPR measure of BOLD activity and *t*-value contrasts. This prevented circularity and overfitting in the sPR method. Each run consisted of six sets of four 16 s blocks. In a given set, each of the first three blocks consisted entirely of exemplars from one of the three categories shown 0.8 s apart. The fourth block was blank to compensate for hemodynamic delay. The order of categories in the first three blocks were pseudo-random such that each category was presented six times per run. While fixating, subjects performed a one-back attention task throughout the runs to ensure alertness. In the one-back task an image was randomly shown twice in a row, signaling the subject to press a button. To localize FFA and PPA in each subject, *t*-values taking into account a 6 s hemodynamic delay were calculated for every voxel in the fMRI volume for the following contrasts: faces vs. inanimate objects and places vs. inanimate objects. FFA and PPA in both left and right hemispheres were defined for the large vein suppressed and magnitude measures of BOLD activity as the largest, contiguous cluster of significant voxels (*t* > 3, *p* < 0.005) in the ventral temporal cortex of each hemisphere.

The scanning protocol consisted of 36 coronal slices collected using standard gradient-echo EPI with voxel size 2.25 × 2.25 × 2.5 mm^3^, FOV 144 × 144 mm^2^, flip angle 72°, TR 2 s, and TE 28 ms. Motion correction was performed using SPM (http://www.fil.ion.ucl.ac.uk/spm), supplemented by additional custom Matlab algorithms. Motion parameters were calculated from the magnitude volumes and then subsequently applied to both magnitude and phase volumes separately for reslicing. Importantly, prior to motion correction of the phase images, large scale phase wrapping due to field inhomogeneities was removed on a slice by slice basis using homodyne filtering (Noll et al., 1991; Reichenbach and Haacke, 2001). For each run and each individual voxel, drift in both magnitude and phase voxel timecourses was removed by fitting a third-degree polynomial. The timecourses were then z-scored (normalized to mean 0.0 and standard deviation 1.0).

#### Quantification of large vein contributions to ROIs

The normalized size of each ROI after large vein suppression was used to quantify the amount of large vein contribution across individual ROIs. Normalized ROI size is defined as the ratio of ROI size with and without large vein suppression:

$$\begin{array}{l} N_{norm}=N_{sPR}∕N_{mag}, \end{array}$$

where *N*_*sPR*_ and *N*_*mag*_ are the number of voxels in a given ROI using sPR and the standard magnitude measure of BOLD activity, respectively. The percentage of voxels with large vein contributions can then be calculated as (1-*N*_*norm*_) ^*^ 100%. The normalized ROI size, rather than the ROI size difference, allows comparison of large vein contributions to ROI size across subjects and should not be confused with absolute (un-normalized) ROI size which is used in subsequent analysis.

#### Correlation between ROI size, FSNR and large vein contributions

**Figure 7:**
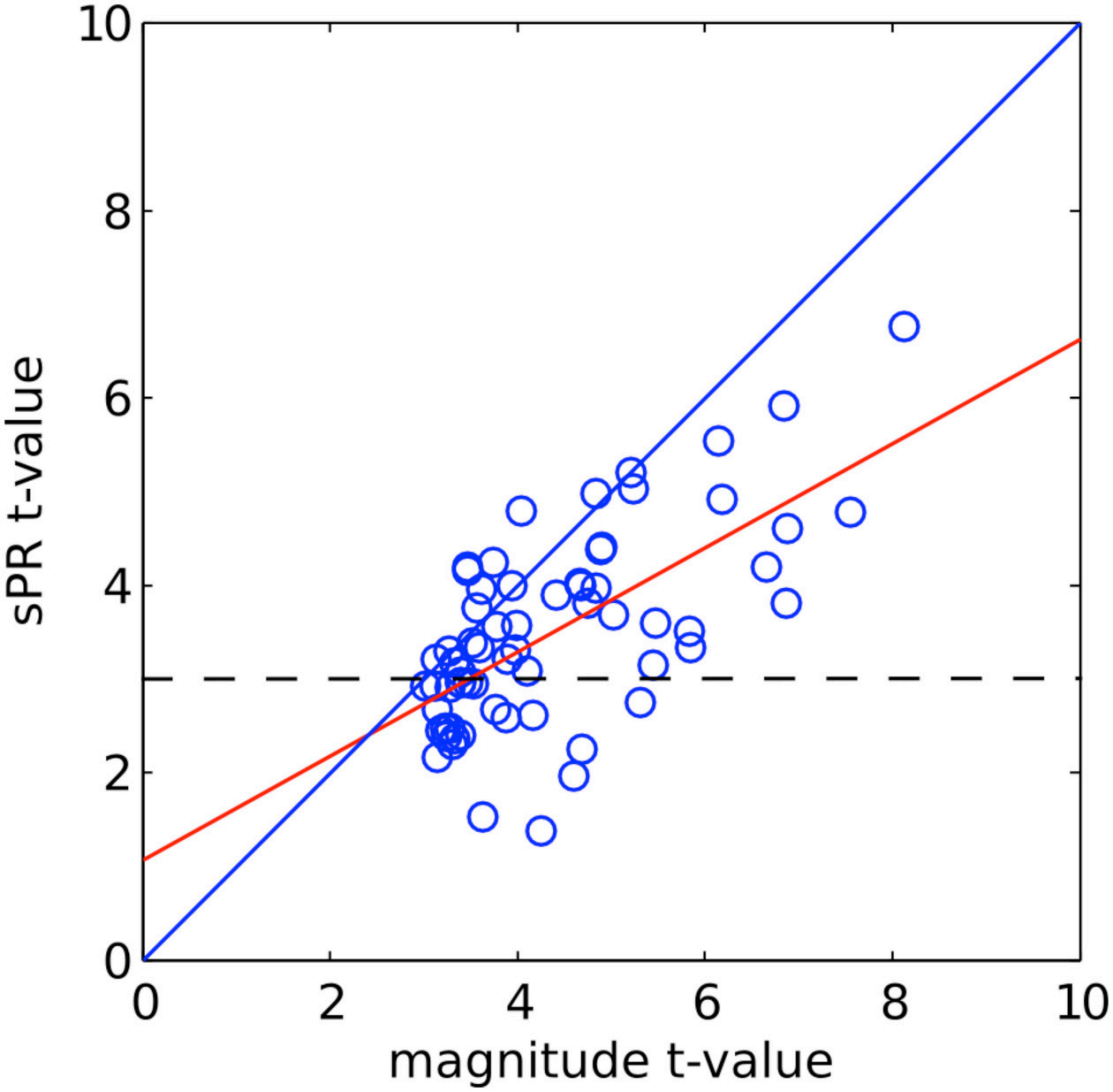
**sPR *t*-values plotted against magnitude *t*-values for right PPA voxels of one typical subject**. The linear fit is shown in red. The dashed line shows the significance level (*t* > 3, *p* < 0.005). Many high magnitude *t*-valued voxels are reduced by large vein suppression but remain significant.

To correlate the amount of large vein contribution to the size and fSNR of individual ROIs, we computed two metrics quantifying the amount of large vein contribution to individual ROIs. Metric 1 is defined as the ROI size difference (measured in number of voxels) without vs. with sPR large vein suppression. This metric measures the number of voxels that are significant (*t* > 3, *p* < 0.005) after large vein suppression. Thus, it does not capture suppression of *t*-values in voxels that remain significant and tends to be more sensitive to suppression in voxels with lower magnitude *t*-values (Figure 7). To be more equally sensitive to suppression of voxels with higher magnitude *t*-values, metric 2 is defined as the difference between mean ROI *t*-values without vs. with sPR large vein suppression. For both metrics, values close to zero indicate no large vein contribution while larger positive values indicate more large vein contributions. The correlations between these two metrics, magnitude ROI size and magnitude ROI fSNR (i.e., without large vein suppression) were calculated (**Figure 10**).

#### Quantification of hemisphere laterality

To quantify the hemisphere laterality of FFA and PPA, an ROI size laterality index and an fSNR laterality index for each subject's FFA and PPA were calculated. For a given subject and ROI, ROI size laterality is defined as:

$$\begin{array}{l} N_{lat}^{ROI}=\left(N_{r}^{ROI}-N_{l}^{ROI}\right)∕\left(N_{r}^{ROI}+N_{l}^{ROI}\right), \end{array}$$

where $N_{r}^{ROI}$ and $N_{l}^{ROI}$ are the number of voxels in an ROI in the right and left hemispheres, respectively. fSNR laterality is similarly defined as:

$$\begin{array}{l} t_{lat}^{ROI}=\left({\bar{t}}_{r}^{ROI}-{\bar{t}}_{l}^{ROI}\right)∕\left({\bar{t}}_{r}^{ROI}+{\bar{t}}_{l}^{ROI}\right), \end{array}$$

where ${\bar{t}}_{r}^{ROI}$ and ${\bar{t}}_{l}^{ROI}$ are the mean *t*-values of an ROI (as defined without large vein suppression) in the right and left hemispheres, respectively. For both indices, positive values indicate right hemisphere laterality and negative values indicate left hemisphere laterality. To determine if hemisphere laterality could be due to a lateral bias in vein size, these indices were calculated with and without sPR large vein suppression.

#### Venograms

Susceptibility weighted venograms were collected and preprocessed using standard methods (Reichenbach and Haacke, 2001). Venograms were collected for ground truth on the location of veins and to insure that sPR large vein suppression was indeed targeting large veins. The 2D gradient-echo venogram scan parameters were: voxel size 1.0 × 1.0 × 1.0 mm^3^, FOV 192 × 192 mm^2^, flip angle 75°, TR 4.21 s, TE 26 ms. The slice prescription covered 96 coronal slices of the most posterior portion of the brain matching the spatial coverage of the fMRI data. Homodyne filtering was done on a slice by slice basis to remove large scale phase wrapping due to field inhomogeneities. Minimum intensity projection was performed in the axial plane on a sliding slab of three slices to match the spatial resolution of the fMRI experiment. Finally, the venograms were manually coregistered to the first functional volume of the fMRI experiment of each subject using custom software.

### Results

#### sPR reveals large vein contributions to ROIs

**Figure 8:**
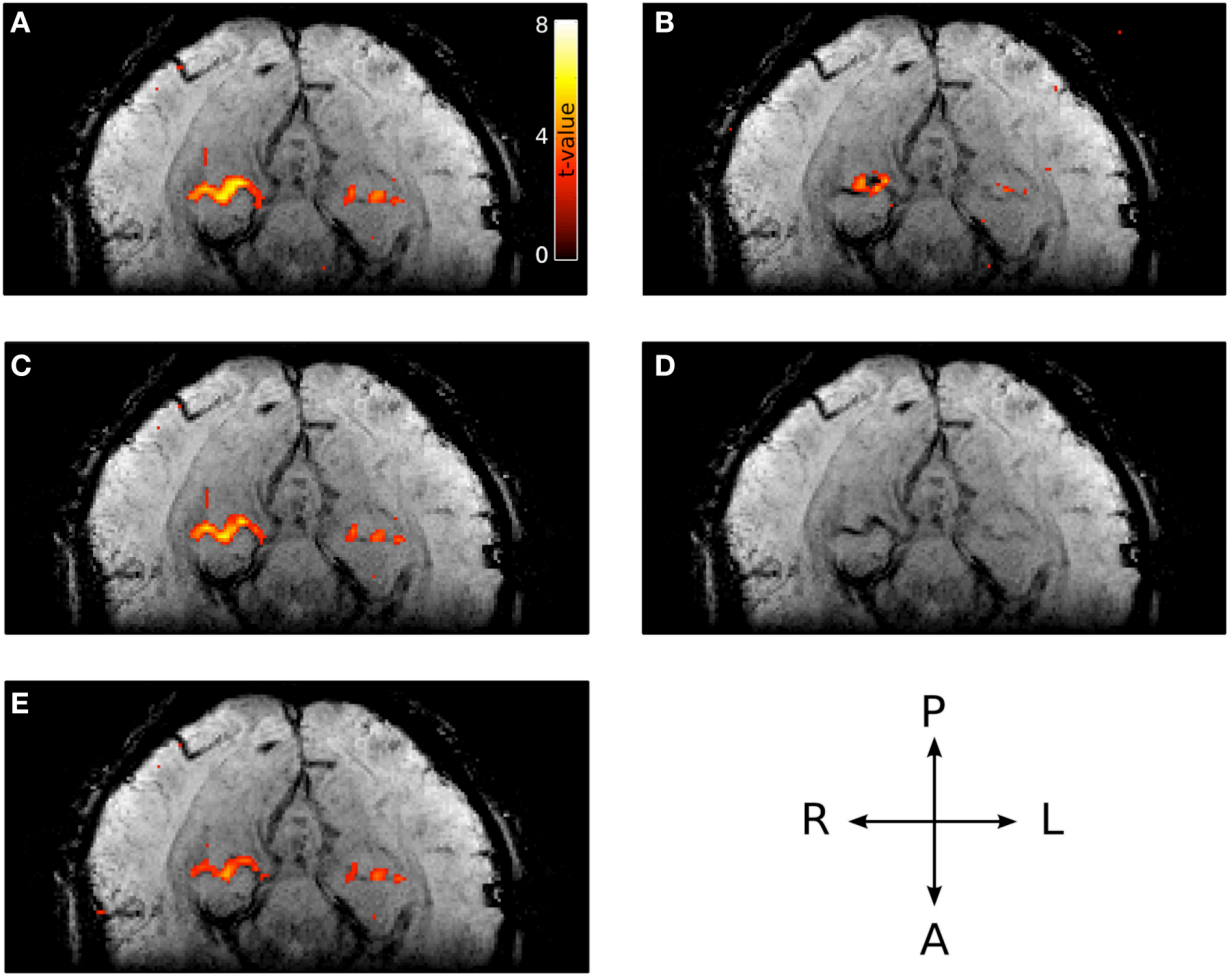
**Typical PPA *t*-values with and without large vein suppression overlaid on the corresponding venogram**. **(A)** Magnitude *t*-values. The majority of right PPA coincides with a large vein. **(B)** Phase *t*-values (absolute value shown to account for opposing susceptibility gradient polarities around the vein). The majority of high phase *t*-valued voxels lie adjacent to the vein. **(C)** Large vein suppressed *t*-values using sPR with a one-voxel-neighborhood (e.g., the original PR method with OLS cost function). In this regime, sPR very minimally suppresses the large vein contributions to right PPA because most of the high phase *t*-values lie adjacent to the vein. **(D)** Corresponding venogram slice. **(E)** Large vein suppressed *t*-values using sPR with the full seven-voxel-neighborhood. In this regime, sPR utilizes the phase component of the BOLD activity from neighboring voxels to correctly suppress the large vein contributions to right PPA. The vein contributing to left PPA is too small to be suppressed using sPR.

Representative *t*-value contrasts for the localization of PPA is shown in Figure 8. The magnitude *t*-values (i.e., without large vein suppression) of the right PPA overlap entirely with a large vein (Figure 8A). This large vein as well as others are shown as the low intensity voxels in the venogram of Figure 8D (Reichenbach and Haacke, 2001). As expected from the phase distribution in and around large veins (Figure 2), the *t*-values generated using the phase data alone can be weak in voxels containing the large vein but strong in voxels adjacent to the vein (Figure 8B). Given this, it is not surprising that using sPR with only a one-voxel-neighborhood (i.e., the original PR method with OLS cost function) yields poor large vein suppression in right PPA (Figure 8C). With the full seven-voxel-neighborhood, sPR is able to utilize the high phase fSNR voxels around the large vein and correctly suppress the *t*-values in right PPA (Figure 8E). This result suggests that consideration of the magnitude and phase fSNR distribution around veins is crucial for the suppression of large vein BOLD activity. For this subject, left PPA appears to lie on a smaller vein. Because the vein is small relative to the size of the voxels scanned, sPR only weakly suppresses the *t*-values in the left PPA of this subject. For completeness, *t*-value contrasts for both FFA and PPA for all subjects are shown in Figures S1–S8.

**Figure 9:**
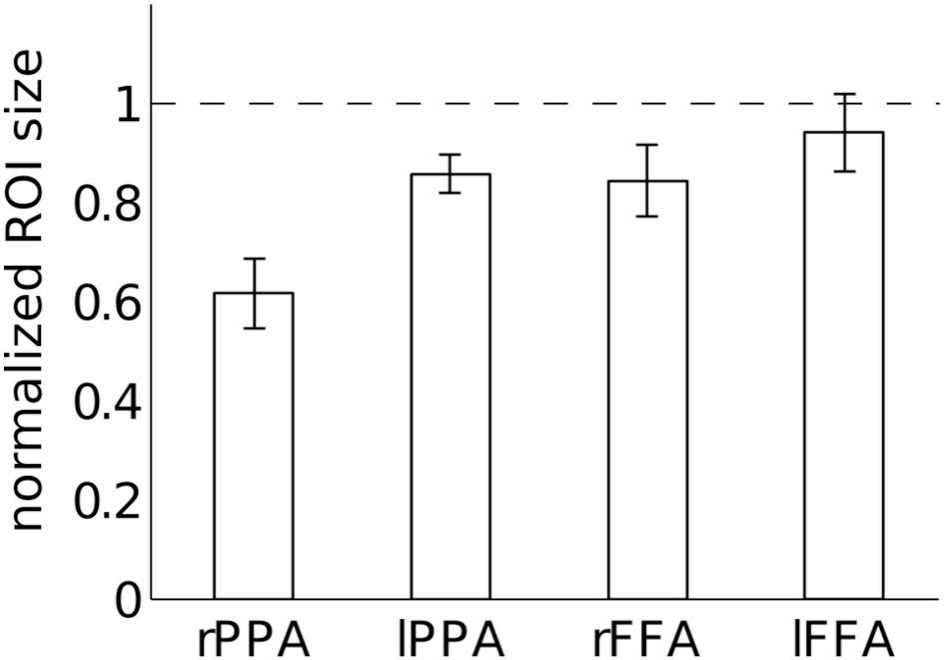
**Quantification of large vein contributions to specific ROIs**. Normalized ROI size after large vein suppression is calculated for each ROI as the ratio of ROI size with vs. without sPR large vein suppression. sPR significantly reduces ROI size for right PPA, left PPA and right FFA (*p* < 0.05). Specifically, 38 ± 7% of right PPA, 14 ± 4% of left PPA, 16 ± 7% of right FFA, and 6 ± 8% of left FFA voxels predominantly reflect signal from large draining veins. Error bars are SEM across subjects.

Figure 9 shows the normalized size of each ROI with relative to without large vein suppression. This normalized measure of ROI size allows us to determine how much smaller specific ROIs are without large vein contributions on average across subjects. sPR large vein suppression significantly reduces the ROI sizes of right PPA, left PPA and right FFA [*T*_(3)_, *p* < 0.05]. Specifically, it is estimated that 38 ± 7% of right PPA, 14 ± 4% of left PPA, 16 ± 7% of right FFA, and 6 ± 8% of left FFA voxels reflect signal predominantly from large draining veins. Notably, large vein contributions to the size of right PPA is significantly greater than the contributions to other ROIs [paired-*T*_(3)_, *p* < 0.05]. Left FFA does not have significant large vein contributions. *F*-tests of these data reveals that the amount of large vein contribution varies significantly across ROIs (*p* < 0.05). These results suggest that the distribution of vein sizes is significantly biased toward specific regions of interest (ROIs).

#### Large vein contributions as a function of ROI size and fSNR

**Figure 10:**
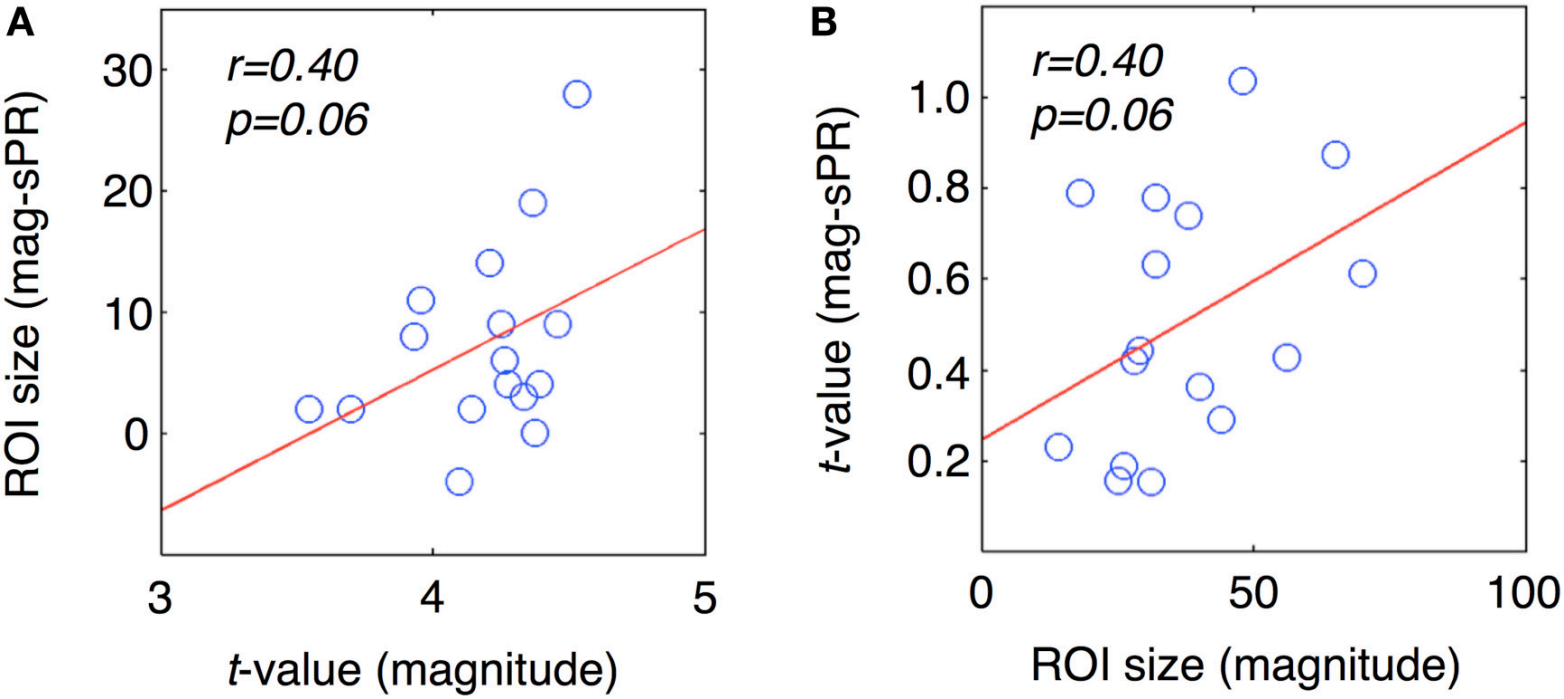
**Correlation between large vein contribution, ROI size, mean *t*-value**. Each data point represents a single ROI from a single subject. **(A)** Large vein contribution metric 1 plotted against mean ROI *t*-value [*r* = 0.40, *T*_(14)_, *p* = 0.06]. Metric 1 is defined as: ROI size without minus with sPR large vein suppression. **(B)** Large vein contribution metric 2 plotted against mean ROI size [*r* = 0.40, *T*_(14)_, *p* = 0.06]. Metric 2 is defined as: mean ROI *t*-value without minus with sPR large vein suppression. These results suggest that ROIs higher in fSNR and larger in size tend to have larger contributions from large veins.

To further determine if semantic area localization is biased toward large veins, we calculated the correlation between large vein contributions, magnitude ROI size and magnitude ROI fSNR (i.e., without large vein suppression). Each ROI of each subject was treated as a separate data point. Figure 10A shows the correlation between large vein contribution metric 1 and mean ROI *t*-value [*r* = 0.40, *T*_(14)_, *p* = 0.06]; with metric 1 defined as: ROI size without minus with sPR large vein suppression. Figure 10B shows the correlation between large vein contribution metric 2 plotted against ROI size [*r* = 0.40, *T*_(14)_, *p* = 0.06]; with metric 2 defined as: mean ROI *t*-value without minus with sPR large vein suppression. These results suggest that across cortical regions and subjects, higher in fSNR and larger in size tend to have larger contributions from large veins. Note metric 1 and metric 2 were not compared to magnitude ROI size and mean magnitude ROI *t*-value, respectively, given that they are not statistically independent by definition.

#### Large vein contributions to hemisphere laterality

**Figure 11:**
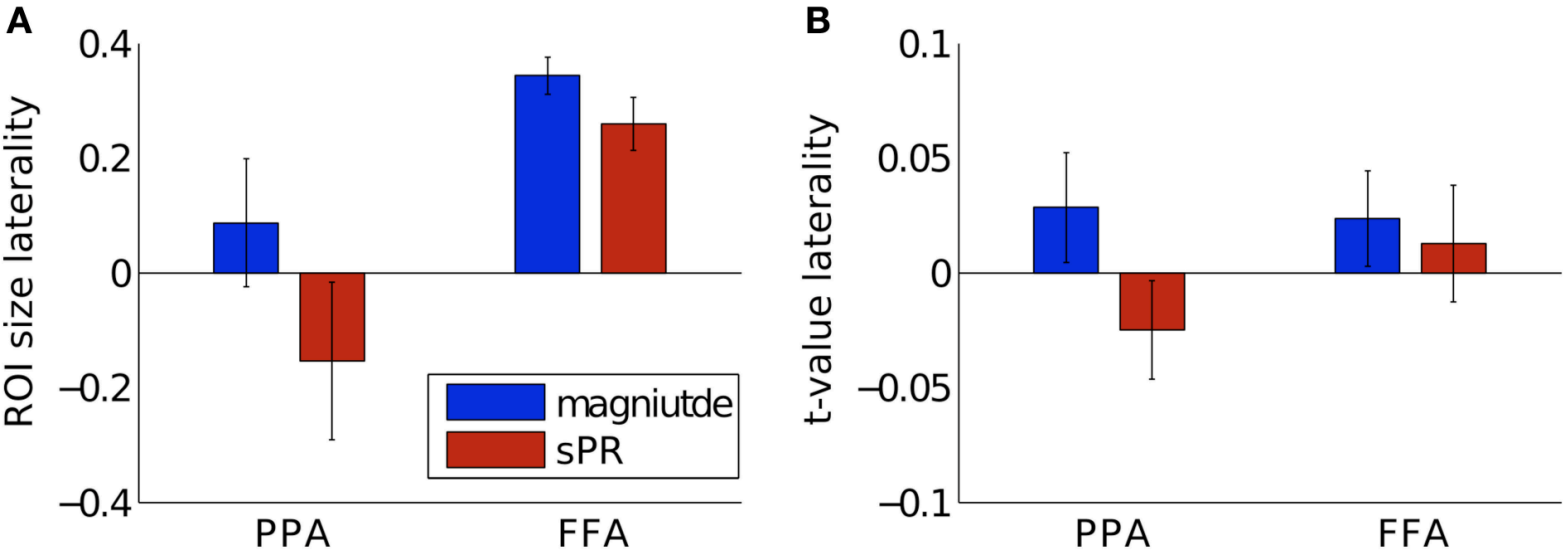
**Hemisphere laterality with and without large vein suppression**. Positive values indicate right hemisphere lateralization while negative values indicate left hemisphere lateralization. **(A)** Size laterality measured as the ratio of hemispheric difference to hemispheric sum of ROI size. Large vein suppression significantly reduces right hemisphere size laterality in PPA [*p* < 0.02, paired-*T*_(3)_] but only to a marginally significantly degree in FFA (*p* = 0.08). **(B)** fSNR laterality measured as the ratio of hemispheric difference to hemispheric sum of mean ROI *t*-value. Large vein suppression significantly reduces right hemisphere fSNR laterality in PPA (*p* < 0.05) but not significantly in FFA (*p* = 0.18). Error bars are SEM across subjects.

To determine if the laterality of semantic representation is biased by larger veins in the right hemisphere, we computed laterality indices of FFA and PPA with and without large vein suppression (Figure 11). Positive laterality values indicate right hemisphere lateralization while negative values indicate left hemisphere lateralization. Consistent with previous studies, without large vein suppression, both PPA and FFA are right hemisphere lateralized in both size ($N_{lat}^{PPA}=0.09$ ± 0.11, $N_{lat}^{FFA}=0.34$ ± 0.03, Figure 11A) and fSNR ($t_{lat}^{PPA}=0.03$ ± 0.02, $t_{lat}^{FFA}=0.02$ ± 0.02, Figure 11B). However, with large vein suppression, PPA is not right but left hemisphere lateralized in size ($N_{lat}^{PPA}$ = −0.15±0.14) and fSNR ($t_{lat}^{PPA}=-0.02\pm 0.02$). These differences in PPA ROI size and fSNR lateralization with vs. without large vein suppression are significant [paired-*T*_(3)_, *p* < 0.02 and *p* < 0.05, respectively]. With large vein suppression, FFA is still right hemisphere lateralized in size ($N_{lat}^{FFA}=0.26\pm 0.05$) and fSNR ($t_{lat}^{FFA}=0.01\pm 0.03$) although less so than without suppression [paired-*T*_(3)_, *p* = 0.08 and *p* = 0.18, respectively]. These results suggest that the degree of right hemisphere laterality of semantic category representation in the human brain can be biased by larger contributions from large veins in the right hemisphere. This is consistent with the tendency for veins to be larger in the right hemisphere (Di Chiro, 1972; Durgun et al., 1993; Ayanzen et al., 2000; Stoquart-ElSankari et al., 2009).

## Discussion

The results of this study challenge the assumption that gradient-echo BOLD fMRI can be used to infer neural differences across cortical regions and across subjects. Previous studies used this assumption to suggest that semantic representation is right hemisphere lateralized and that semantic category areas are rare (for review see Reddy and Kanwisher, 2006). Given the large vein bias demonstrated in this study, these well known properties of semantic category representation should be reevaluated.

Our results on the relationship between ROI size, fSNR and large vein contributions provide important insight into the lateralization of semantic category representation (Kanwisher et al., 1997; Epstein and Kanwisher, 1998; Downing et al., 2006; Spiridon et al., 2006). Finding that right PPA has more large vein contributions than left PPA (Figure 9) suggests that the degree of right hemisphere laterality of PPA reported in prior studies may be biased by hemispheric differences in vein size. Calculation of PPA laterality indices with large vein suppression reveals that PPA may actually be left hemisphere lateralized (Figure 11). FFA was also found to be less right hemisphere lateralized after large vein suppression. Together, these results suggest that researchers using gradient-echo BOLD fMRI to infer laterality of semantic representation should be careful to account for hemispheric differences in vein size (Di Chiro, 1972; Durgun et al., 1993; Ayanzen et al., 2000; Stoquart-ElSankari et al., 2009).

Furthermore, our results provide important insight into the rarity of semantic category areas (Downing et al., 2006; Spiridon et al., 2006). As mentioned previously, studies of FFA often report subjects with no detectable left FFA. Our finding that left FFA has no significant large vein contribution suggests that semantic category areas difficult to detect, such as left FFA, may not reflect absence of neural representation. Instead, difficulty or inability to detect certain ROIs using gradient-echo BOLD fMRI may reflect a vascular bias away from those ROIs. This would not be surprising given recent physiological evidence for the existence of left FFA. One study reported that over 90% of visually responsive neurons responded selectively to faces in a face selective region in the left hemisphere of a macaque monkey (Tsao et al., 2006). Face-selective neurons were also equally numerous and equally face-selective in both hemispheres. Another study (Tsao et al., 2008) showed strong bilateral face-selective activity in 9 of 10 macaque monkeys using contrast-enhanced fMRI. Importantly, this fMRI method did not depend on the gradient-echo BOLD contrast which is biased toward large veins. Instead, it used monocrystalline iron oxide nanoparticles (MION) to enhance cerebral blood volume functional contrast which is more specific to the microvasculature (Mandeville and Marota, 1999; Vanduffel et al., 2001). Together, these results suggest that the rarity of semantic category areas reported in previous studies may reflect large vein bias toward specific ROIs.

The results of this study emphasizes the fact that BOLD fMRI only indirectly measures neural activity through the cortical vasculature. Current understanding of cortical vasculature organization with respect to underlying neurons is poor with relatively few experimental studies on this subject (Gardner, 2010; Harel et al., 2010). Some studies have shown that functional grouping of microvasculature is correlated with functional grouping of neurons in the rat barrel cortex (Woolsey et al., 1996; Berwick et al., 2008). The relationship between the functional organization of microvasculature and neurons has been further investigated by studies demonstrating the plasticity of cortical capillary density across several weeks (Pichiule and Lamanna, 2002; LaManna et al., 2004). Other studies proposed that venous functional organization exists on a larger scale across cortical columns (Gardner, 2010; Kriegeskorte et al., 2010; Op de Beeck, 2010; Shmuel et al., 2010). These studies suggest that larger venules and veins receive drainage from similarly tuned neurons across several cortical columns. This would allow low resolution BOLD fMRI to resolve stimulus information represented at the sub-voxel scale (e.g., stimulus orientation).

The results of this study suggest that functional organization between venous vasculature and neurons (referred to as venous functional organization) may exist, and that it may vary considerably at the scale of cortical areas. Previous studies have reported greater vascularization in primary visual cortex (V1) than in area V2 (Duvernoy et al., 1981; Zheng et al., 1991; Logothetis and Wandell, 2004). However, this is the first study that demonstrates vascular differences across semantic category areas.

By using methods that take into account or remove large vein contributions from the BOLD signal, the problems of large vein bias can be mitigated. Such methods will not only improve the point spread function (e.g., effective spatial resolution of the BOLD signal), which is on the order of millimeters (Turner, 2002; Parkes et al., 2005; Shmuel et al., 2007), but also large scale biases due to venous functional organization, which can be on the order of several centimeters or opposing hemispheres as shown in this study. Future use of methods like sPR may reveal new semantic category areas previously obscured by large vein bias and allow for more complete functional characterization of visual cortex. Fortunately, advances in fMRI methodology including the sPR method presented here can provide robust and efficient suppression of BOLD activity from large veins (Yacoub et al., 2001; Duong et al., 2003; Vanzetta et al., 2004; Hulvershorn et al., 2005).

While prior studies have shown evidence for reduced large vein bias at higher magnetic field strengths due to shorter venous blood T2^*^ (Gati et al., 1997; Yacoub et al., 2001), the majority of fMRI studies at higher field, which use gradient-echo BOLD fMRI, will still contain significant amounts of large vein bias (Geißler et al., 2013). This is because while higher field strengths can reduce intravascular contributions to the BOLD signal, the extravascular contributions still dominate in T2^*^-weighted gradient-echo BOLD fMRI (Duong et al., 2003; Hulvershorn et al., 2005). Thus, T2-weighted spin-echo techniques have traditionally been used at higher field strengths for both intra- and extra-vascular venous suppression at the cost of the lower contrast to noise ratio (CNR) inherent in T2 contrast (Yacoub et al., 2001; Duong et al., 2003; Hulvershorn et al., 2005). Importantly, sPR can suppress both intra- and extra-vascular contributions regardless of field strength while also preserving the high T2^*^ CNR by utilizing the freely available (but unfortunately, often unknowingly discarded) phase component of the gradient-echo BOLD signal. Given that venous susceptibility phase changes scale linearly with field strength (Ogawa et al., 1993a,b), future sPR studies at higher field and higher spatial resolution (for reduced partial voluming) will be even more efficient at suppressing large vein bias which will be important for furthering our understanding of semantic category representation in the human brain.

## Conclusions

We presented sPR as a novel, robust and unbiased method for suppressing large vein contributions to gradient-echo BOLD activity. By measuring ROI size and fSNR with and without large vein suppression, the amount of large vein contribution to semantic category areas FFA and PPA was quantified. ROIs larger in size and higher in fSNR tended to have more large vein contributions. These results support the hypothesis that the apparent paucity and laterality of semantic category areas reflect bias in the distribution of vein size toward specific ROIs. We are reminded that it is important to account for the cortical vasculature when interpreting results of studies using gradient-echo BOLD fMRI and that saving of the freely available phase images can help in this regard.

### Conflict of interest statement

The authors declare that the research was conducted in the absence of any commercial or financial relationships that could be construed as a potential conflict of interest.
