# Supplementary material for: Using a novel source-localized phase regressor technique for evaluation of the vascular contribution to semantic category area localization in BOLD fMRI
Source: Front Neurosci. 2015 Nov 3;9:411. doi: 10.3389/fnins.2015.00411 (PMC4630295; doi:10.3389/fnins.2015.00411)
Supplement: Supplementary file 2 [file DataSheet2.DOCX]

Supplementary Figures:


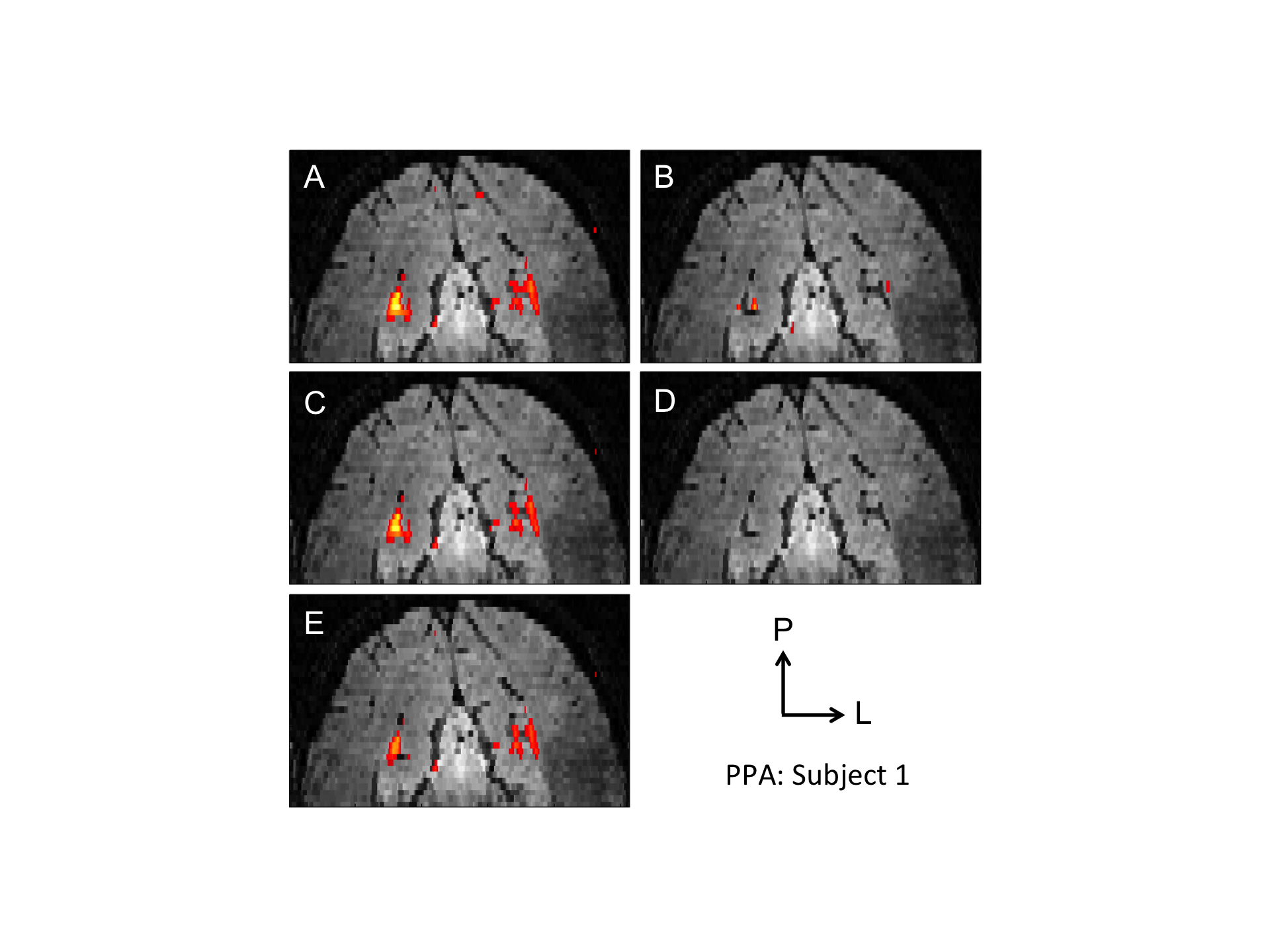
Figure S1. PPA *t*-values with and without large vein suppression overlaid on the corresponding venogram for Subject 1. Format is the same as in Figure 8.


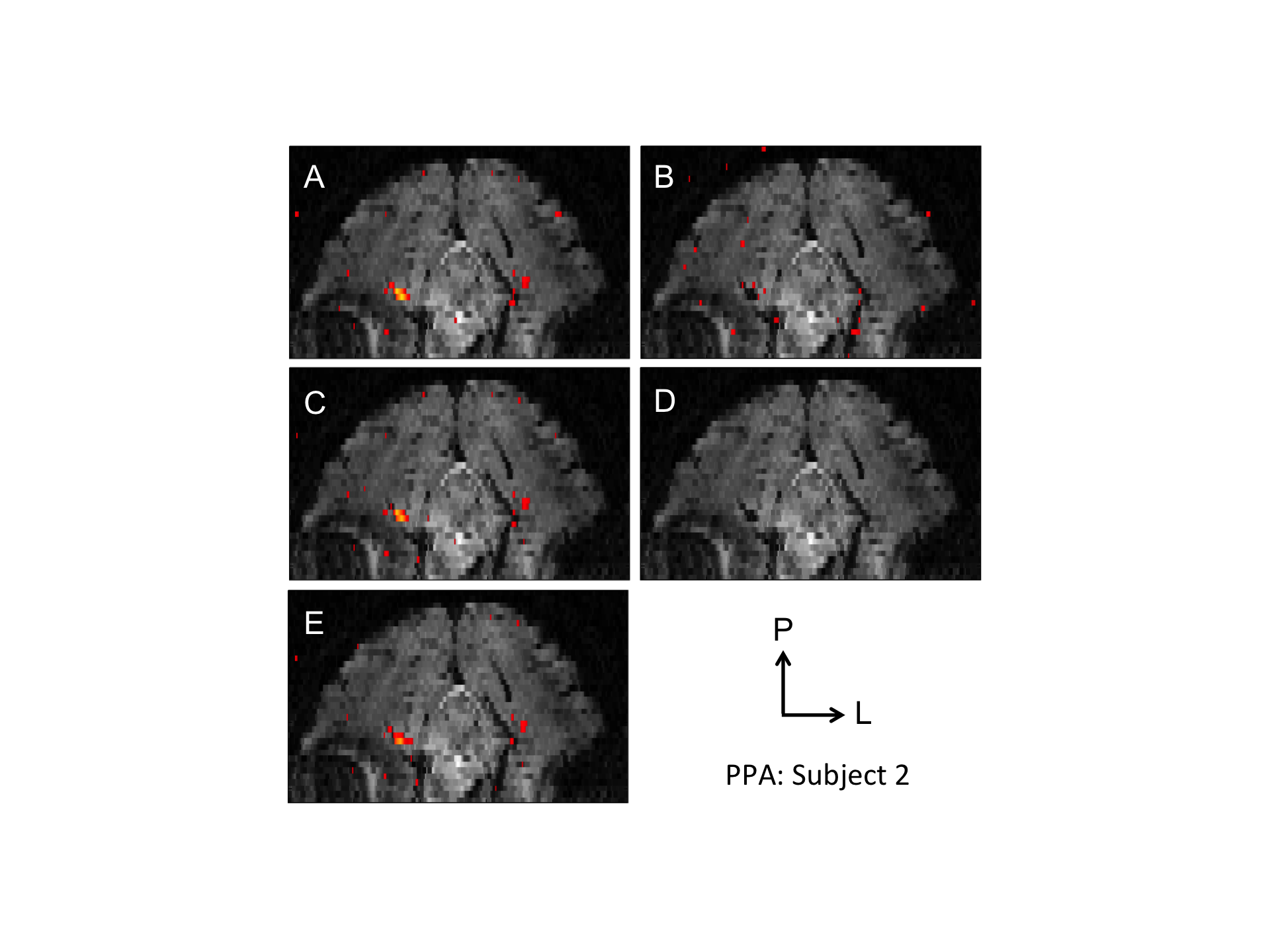
Figure S3. PPA *t*-values with and without large vein suppression overlaid on the corresponding venogram for Subject 3. Format is the same as in Figure 8.


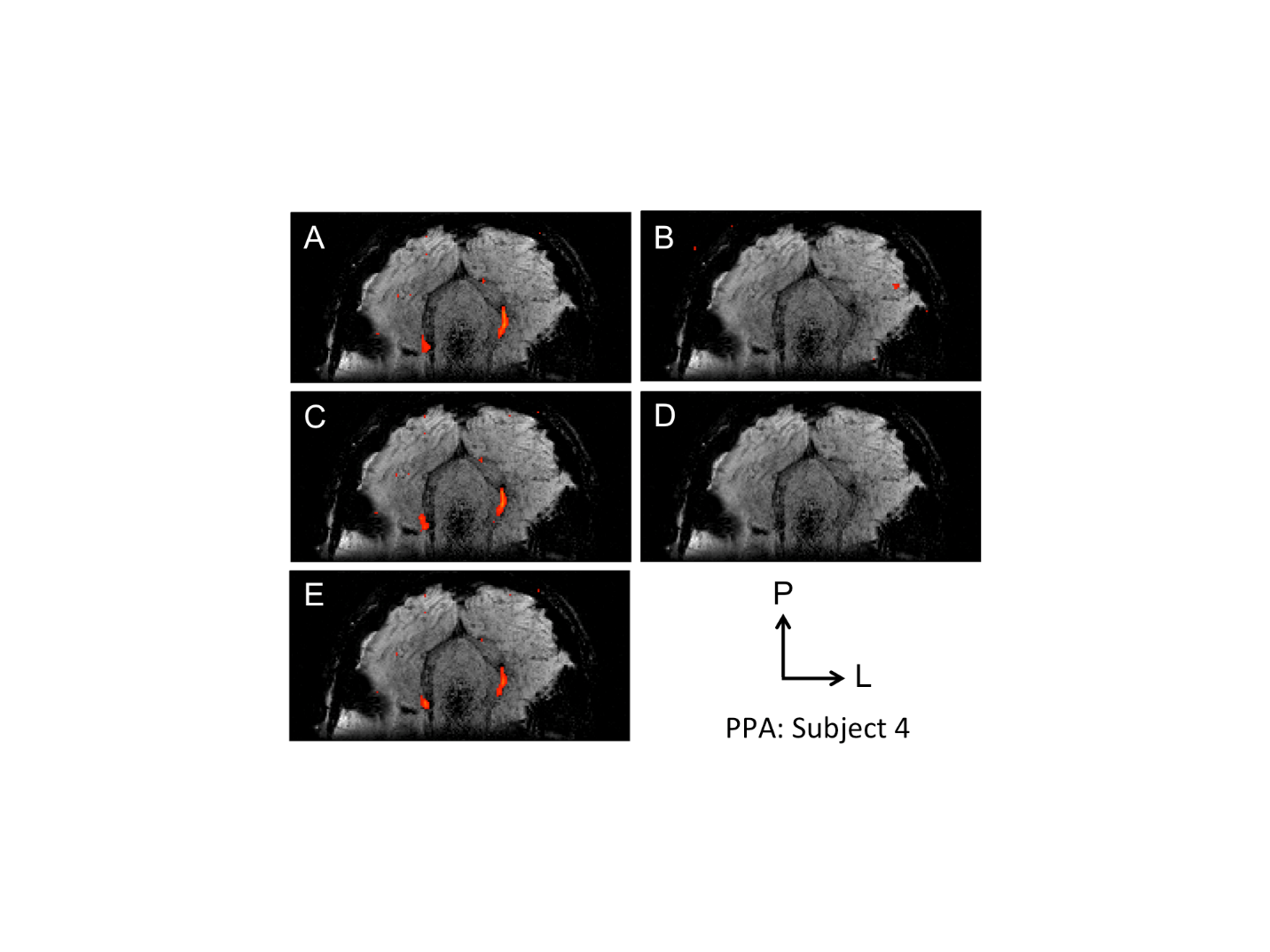
Figure S3. PPA *t*-values with and without large vein suppression overlaid on the corresponding venogram for Subject 3. Format is the same as in Figure 8.


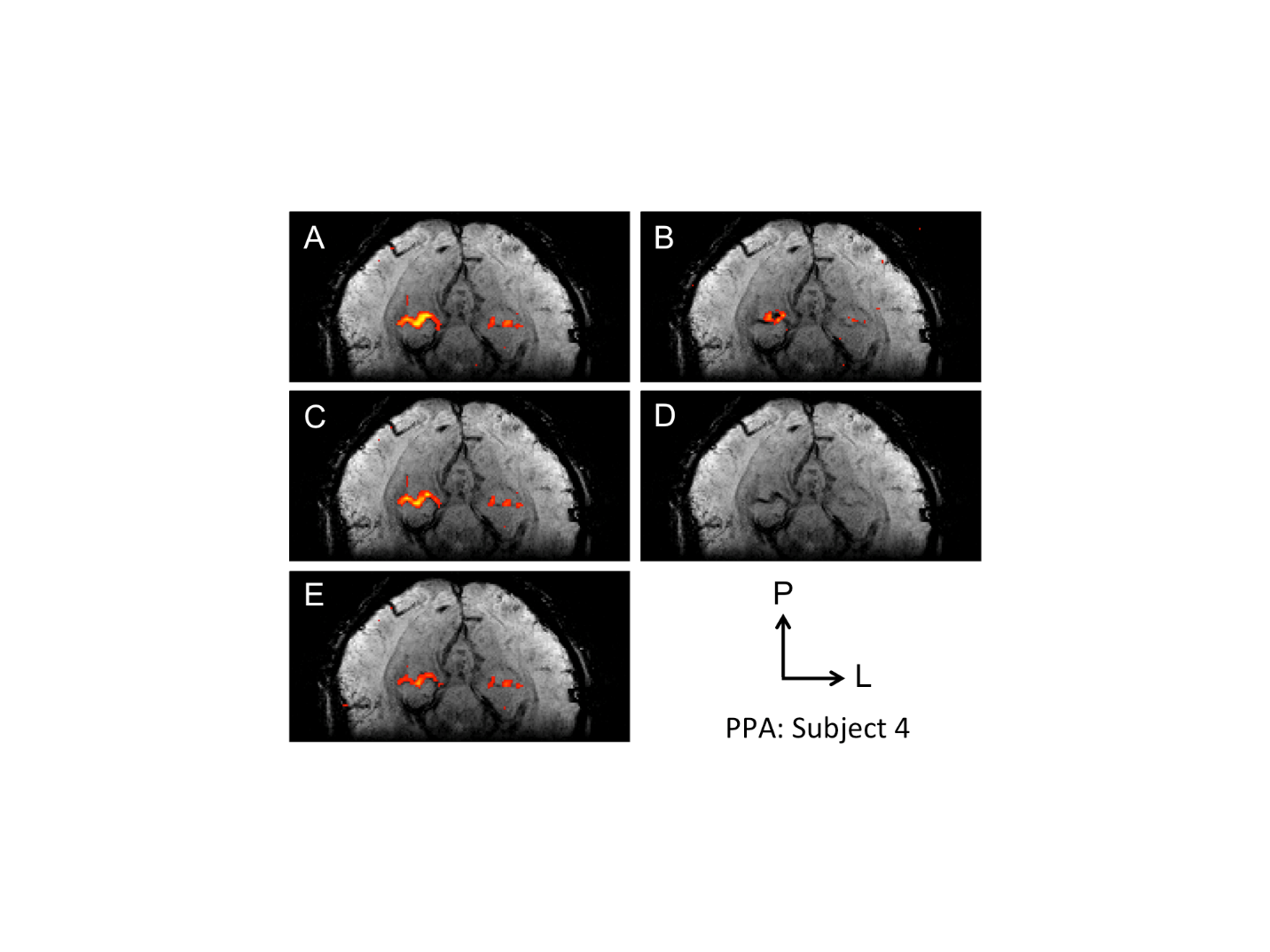
Figure S4. PPA *t*-values with and without large vein suppression overlaid on the corresponding venogram for Subject 4. Format is the same as in Figure 8.


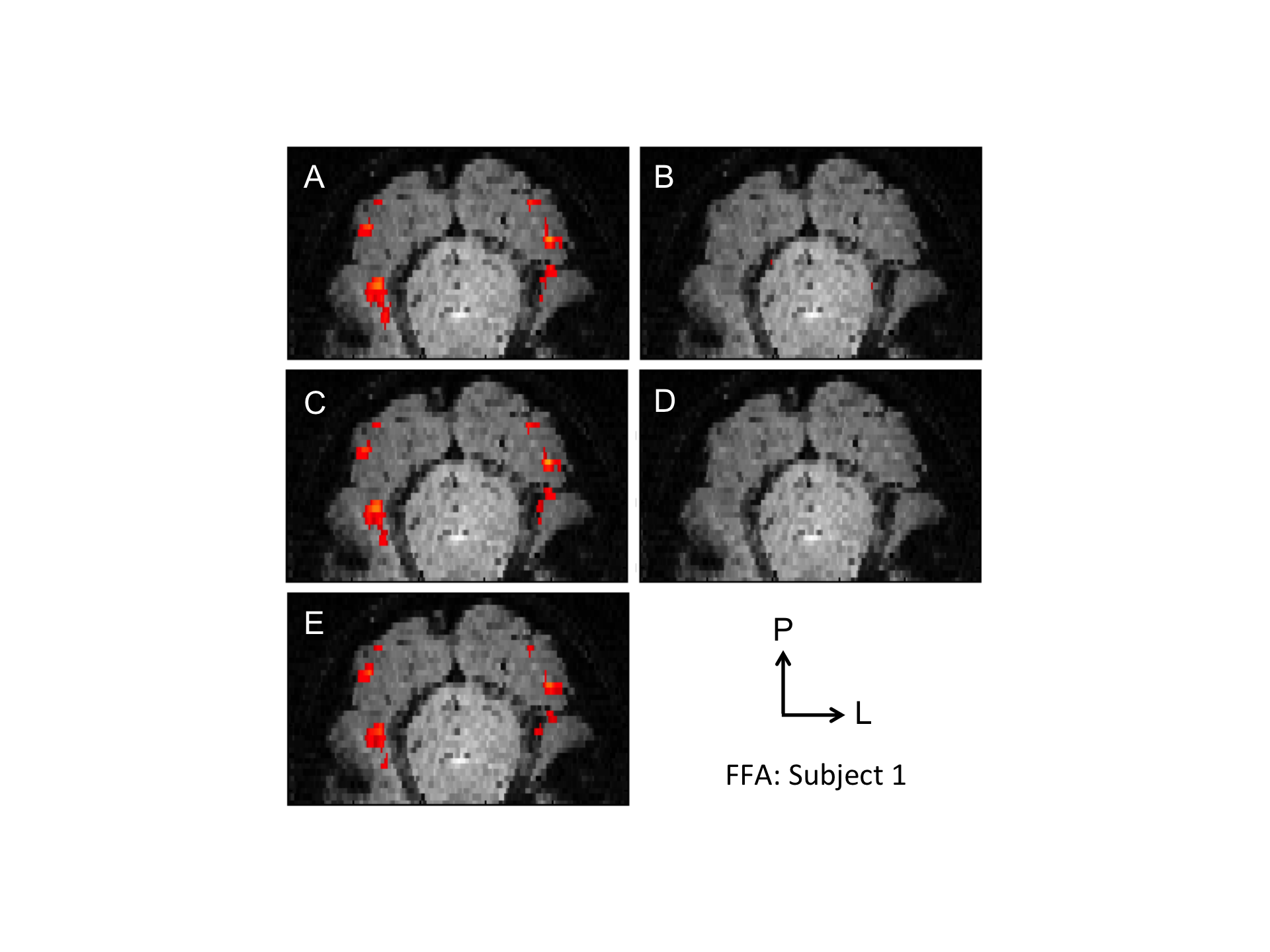
Figure S5. FFA *t*-values with and without large vein suppression overlaid on the corresponding venogram for Subject 1. Format is the same as in Figure 8.


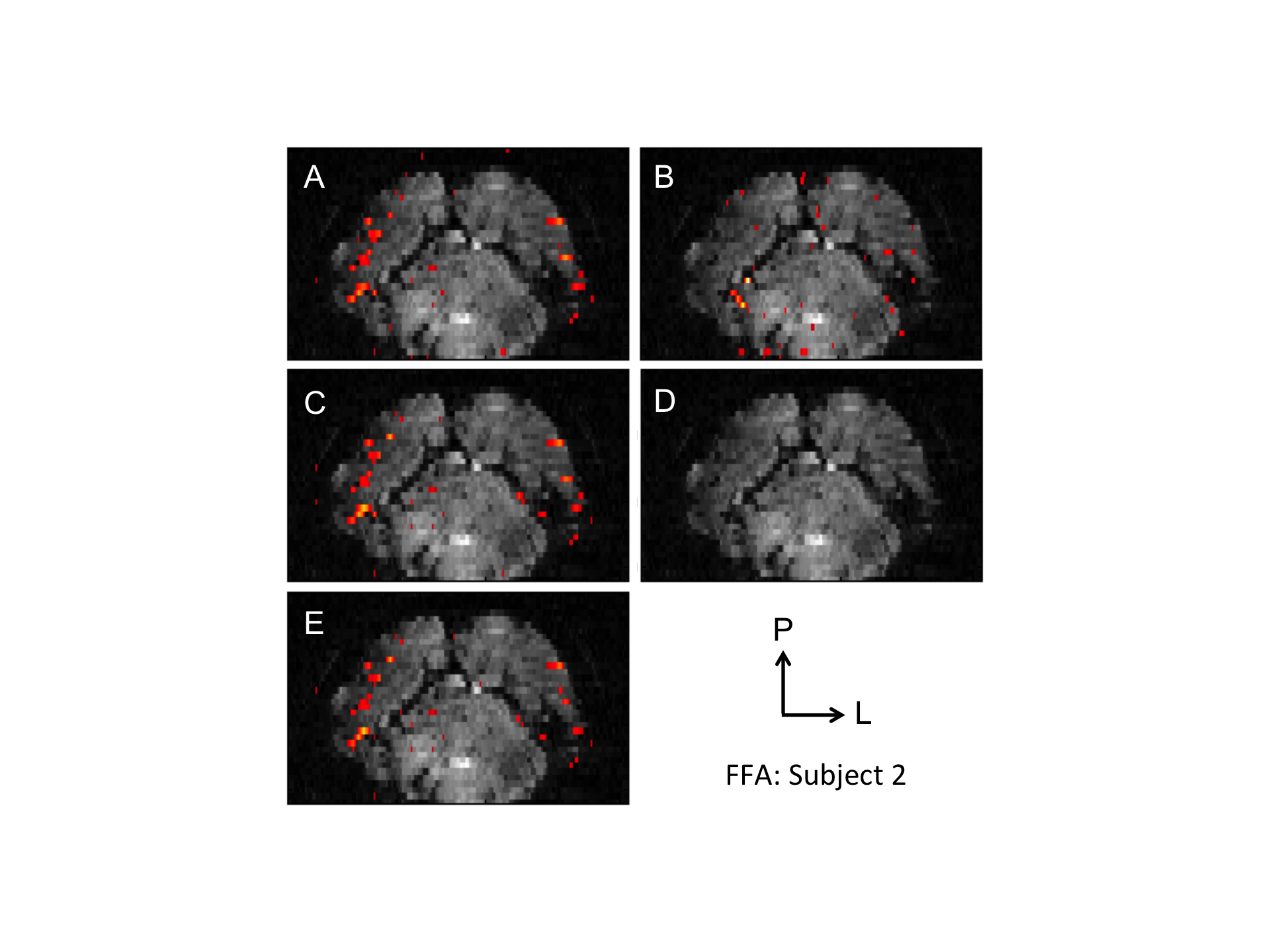
Figure S6. FFA *t*-values with and without large vein suppression overlaid on the corresponding venogram for Subject 2. Format is the same as in Figure 8.


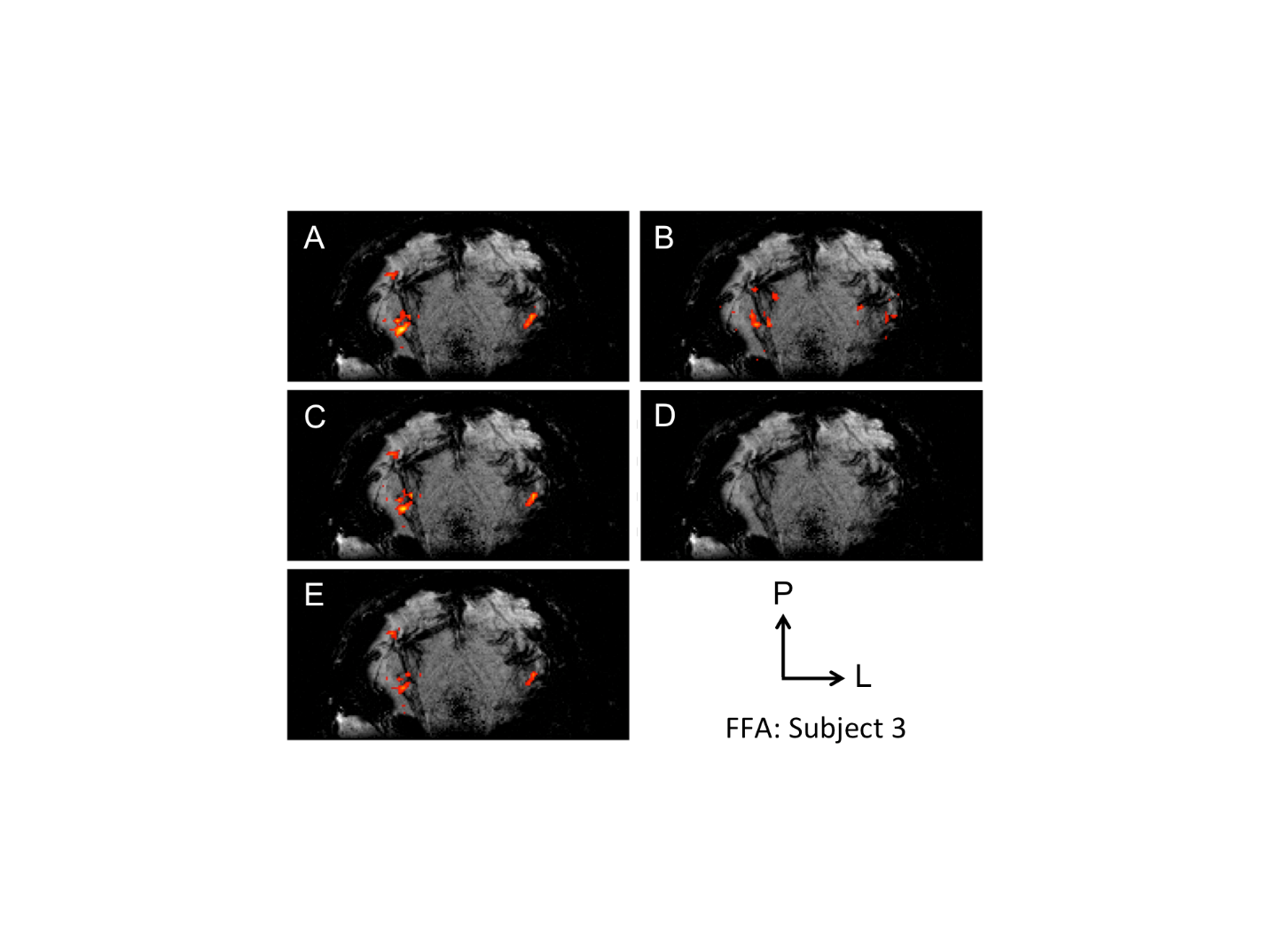
Figure S7. FFA *t*-values with and without large vein suppression overlaid on the corresponding venogram for Subject 3. Format is the same as in Figure 8.


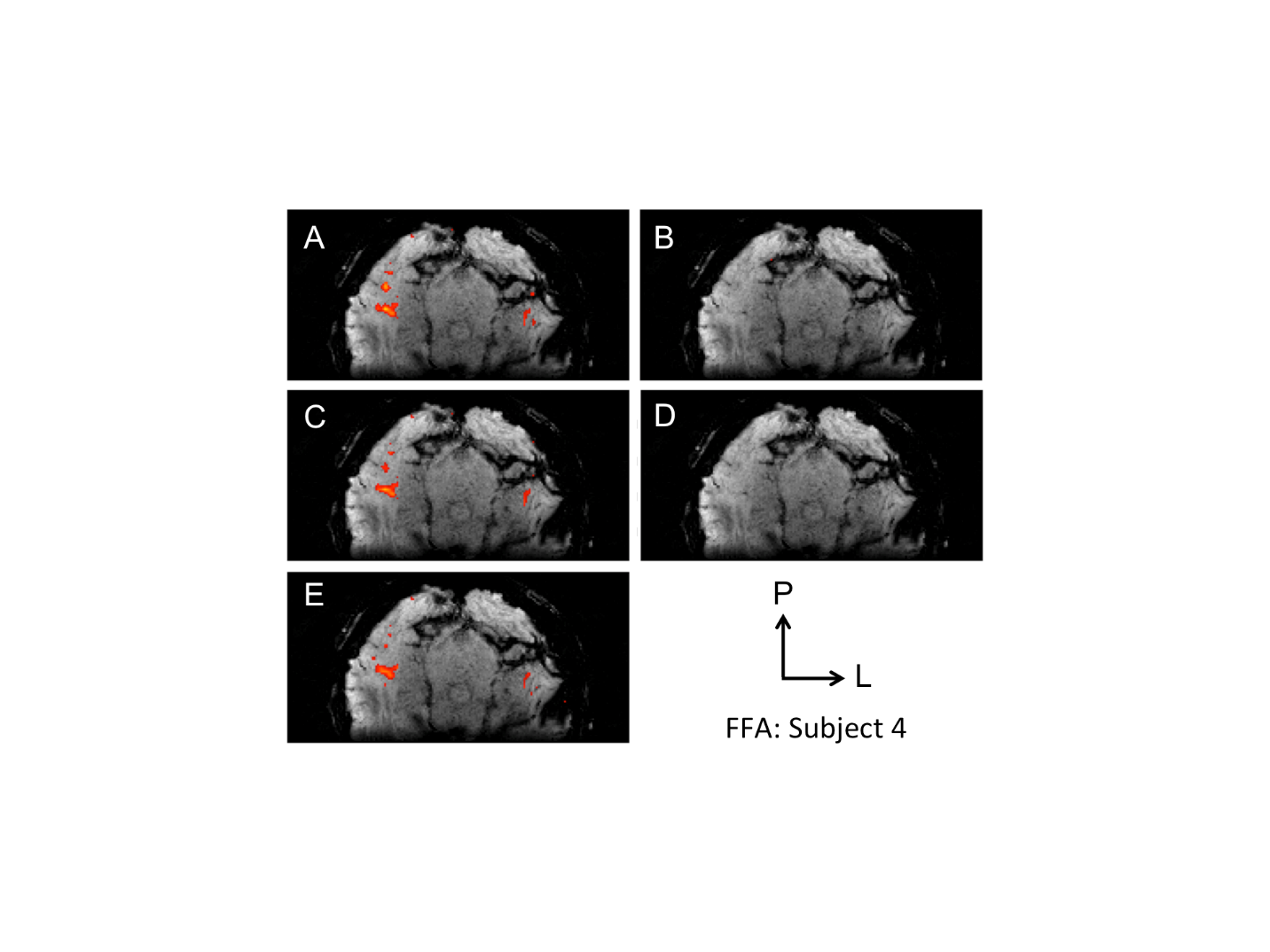
Figure S8. FFA *t*-values with and without large vein suppression overlaid on the corresponding venogram for Subject 4. Format is the same as in Figure 8.
